# Scale Development for Environmental Perception of Public Space

**DOI:** 10.3389/fpsyg.2020.596790

**Published:** 2020-11-23

**Authors:** Robbie Ho, Wing Tung Au

**Affiliations:** ^1^Division of Social Sciences, Humanities and Design, College of Professional and Continuing Education, The Hong Kong Polytechnic University, Hung Hom, Hong Kong; ^2^Department of Psychology, The Chinese University of Hong Kong, Shatin, Hong Kong

**Keywords:** scale development, environmental perception, public place, public space, urban

## Abstract

We developed a psychometric scale for measuring the subjective environmental perception of public spaces. In the scale development process, we started with an initial pool of 85 items identified from the literature that were related to environmental perception. A total of 1,650 participants rated these items on animated images of 12 public spaces through an online survey. Using principal component analyses and confirmatory factor analyses, we identified two affective factors (*comfort* and *activity*) with 8 items and six cognitive factors (*legibility*, *enclosure*, *complexity*, *crime potential*, *wildlife*, and *lighting*) with 22 items. These eight factors represent the core attributes underlying environmental perception of public spaces. Practicality of the scale and limitations of the study are also discussed.

## Introduction

### Need for a Tool That Measures Environmental Perception of Public Spaces

This paper reports the development of a psychometric scale for measuring the subjective environmental perception of public spaces. Public spaces refer to “*open, publicly accessible places where people go for group or individual activities… Some are under public ownership and management, whereas others are privately owned but open to the public*” (Carr et al., 1992, p. 50). Understanding human experience of public spaces is a critical domain in urban studies. Quality of the environment in which people live, according to Pacione (2003), constitutes an important aspect to their quality of life. There are objective and subjective indicators to determine the quality of public spaces. Borrowing Pacione’s words, objective measurements capture “*the city on the ground*” whereas subjective perception captures “*the city in the mind*” (p. 20). Subjective perception is a personal interpretation of an objective situation (Pacione, 2003, p. 21). Such differentiation between the objective and the subjective is echoed by van Kamp et al. (2003); thinking about the quality of an environment should not be dictated by the objective condition of that environment. It is also suggested that subjective perception of environmental quality allows us to gain insight into people’s satisfaction and preferences about places (Pacione, 2003; van Kamp et al., 2003). There has been empirical support for the association between people’s perceived quality of public spaces and their residential satisfaction (Aiello et al., 2010) as well as sense of community (Francis et al., 2012). The need for a standard tool that measures subjective perception of public spaces is called for in recent studies. Legendre and Gómez-Herrera (2011) observed developmental changes in children’s use of and independent access to public spaces. As children grew up, they increased their use of and independent access to public spaces. These findings raise the question on what aspects of public spaces could have accounted for the developmental changes in how children use and access public spaces. In another study, Valera-Pertegas and Guàrdia-Olmos (2017) found that women tended to report higher level of perceived insecurity about public spaces than men. Again, these findings open questions regarding how the same objective public spaces could be subjectively perceived by women and men differently. In order to answer these questions, a comprehensive tool for measuring subjective perception of public spaces is needed.

Psychometric scale is a common practice for capturing people’s subjective perception or experiences about a variety of environmental topics, e.g., connectedness to nature, environmental attitude, ecological behavior, and place attachment. Connectedness to nature encompasses people’s perception about their identification, love, and care for nature (Mayer and Frantz, 2004; Perkins, 2010; Martin and Czellar, 2016). Environmental attitude is a broad category of people’s attitudes and beliefs related to their willingness and intention to take environmental actions, support for conservation interventions or policies, belief in environmental threats, etc. (Homburg et al., 2007; Milfont and Duckitt, 2010; Li and Monroe, 2018; Walton and Jones, 2018; Cakmak, 2020). Ecological behavior refers to actual behaviors such as energy conservation, waste avoidance, education in environmental issues, etc. (Kaiser et al., 2007; Alisat and Riemer, 2015; O’Brien et al., 2018). Place attachment takes care of people’s self-identification with and behavioral dependence on a place (Raymond et al., 2010). Furthermore, there are scales that are evaluative in nature. For example, perceived restorativeness captures the extent to which an environmental setting provides its users with the opportunity to relax and temporarily take a break from their daily stressors (Laumann et al., 2001; Pals et al., 2009). Visitability refers to the extent to which people evaluate a place as friendly for visit (Abdulkarim and Nasar, 2014a, b). There are numerous studies that examined environmental perception of public spaces in particular (Nasar and Cubukcu, 2011; Lindal and Hartig, 2013, 2015; Abdulkarim and Nasar, 2014a, b; Motoyama and Hanyu, 2014; Rašković and Decker, 2015; Nasar and Bokharaei, 2017a, b); however, those studies often only focused on particular aspects of public spaces and their operationalizations of environmental perception were inconsistent. For example, Motoyama and Hanyu (2014) examined only the visual properties of public spaces (i.e., brightness, coherence, complexity, legibility, naturalness, nuisance elements, spaciousness, typicality, and upkeep), whereas Nasar and Cubukcu (2011) were only interested in the mystery (the promise of further information) and surprise (the mismatch from one’s expectations) of public spaces. Existing literature does not provide a standard tool for measuring the environmental perception of public spaces. The current paper fills in the research gap and unifies the core attributes underlying environment perception of public spaces.

### Affective and Cognitive Domains of Environmental Perception

Environmental perception can be divided into *affective* and *cognitive* domains (Nasar, 1994; Aiello et al., 2010). In Nasar’s (1994) model of responses to urban aesthetics, affective and cognitive domains encompass, respectively, the interpreted (e.g., character and atmosphere) and the physical (e.g., size and proportion) attributes of a given urban design. We employ the affective-cognitive distinction to organize psychological constructs related to environmental perception of public spaces. Using keywords “*urban*,” “*public space/place*,” and “*environmental perception/experience/ appraisal/preference/attributes*,” we identified from the literature 20 publications dated from 1980 onward that operationalized environmental perception with self-report items (Russell and Pratt, 1980; Nasar, 1983; Herzog, 1992; Strumse, 1994; Herzog and Gale, 1996; Hanyu, 1997; Herzog and Leverich, 2003; Herzog and Bryce, 2007; Yatmo, 2009; Yüksel, 2009; Nasar and Terzano, 2010; Nasar and Cubukcu, 2011; Ho et al., 2020; Chiang et al., 2014; Motoyama and Hanyu, 2014; Pals et al., 2014; Rašković and Decker, 2015; Nasar and Bokharaei, 2017a, b; van Rijswijk and Haans, 2018). A quarter of those publications explicitly state whether they examined the affective or the cognitive domain of environmental perception. Russell and Pratt (1980) focused entirely on affective appraisals including activity (or arousal), excitement, pleasantness, and relaxation. Herzog (1992) and Strumse (1994) conducted cognitive analysis of visual properties such as coherence, complexity, legibility, and spaciousness. Hanyu (1997) and Motoyama and Hanyu (2014) incorporated constructs from both domains. Although the other 15 publications do not specify which domain(s) of environmental perception they were examining, their constructs could clearly be classified as either affective or cognitive. The 20 publications are organized in Table 1; four of them examined affective constructs, seven cognitive, and nine a combination of both. Note that the constructs overlapped; 10 were unique in the affective domain and 27 were unique in the cognitive domain. Affective constructs included *activity*, *aesthetics*, *crime rate*, *desirable living place*, *excitement*, *interest*, *pleasantness*, *preference*, *relaxation*, and *safeness*. Cognitive constructs included *age*, *brightness*, *building care*, *clarity*, *coherence*, *complexity*, *composition*, *danger*, *enclosure*, *landmarks*, *legibility*, *lighting*, *mystery*, *naturalness*, *nature care*, *nuisance elements*, *perceived crowding*, *refuge*, *safety*, *situational concern*, *spaciousness*, *surprise*, *typicality*, *uniform lighting*, *upkeep*, *vehicles*, and *visibility*. In the affective domain, pleasantness appeared most frequently, followed by aesthetics, excitement, preference, and relaxation. In the cognitive domain, complexity appeared most frequently, followed by coherence, legibility, mystery, perceived crowding, and safety. This body of works provides us with the materials for scale development.

**TABLE 1:** Constructs of environmental perception.

	Affective Domain										Cognitive Domain																										
	Activity	Aesthetics	Crime Rate	Desirable Living Place	Excitement	Interest	Pleasantness	Preference	Relaxation	Safeness	Age	Brightness	Building Care	Clarity	Coherence	Complexity	Composition	Danger	Enclosure	Landmarks	Legibility	Lighting	Mystery	Naturalness	Nature Care	Nuisance Elements	Perceived Crowding	Refuge	Safety	Situational Concern	Spaciousness	Surprise	Typicality	Uniform Lighting	Upkeep	Vehicles	Visibility
Chiang et al., 2014																*							*							*							*
Hanyu, 1997	*				*	*	*		*	*		*			*	*					*		*	*		*					*		*	*		*	
Herzog, 1992											*				*	*			*		*		*					*			*		*				
Herzog and Bryce, 2007															*	*		*			*	*	*														*
Herzog and Gale, 1996											*		*		*	*							*	*	*												
Herzog and Leverich, 2003															*	*	*			*	*		*								*						
Ho et al., 2020																											*										
Motoyama and Hanyu, 2014	*	*			*	*	*	*	*	*		*			*	*					*			*		*					*		*		*		
Nasar, 1983			*	*		*	*							*	*	*			*				*	*		*											
Nasar and Bokharaei, 2017a		*			*					*																					*						
Nasar and Bokharaei, 2017b	*	*			*		*		*	*																											
Nasar and Cubukcu, 2011						*		*															*									*					
Nasar and Terzano, 2010							*																														
Pals et al., 2014		*					*	*							*																						
Rašković and Decker, 2015		*																																			
Russell and Pratt, 1980	*				*		*		*																												
Strumse, 1994								*			*				*	*					*		*								*						*
van Rijswijk and Haans, 2018																						*							*								
Yatmo, 2009								*																					*								
Yüksel, 2009		*					*	*																			*										

### Association Between Environmental Perception and Preference About Public Spaces

Environmental perception can affect people’s satisfaction and preferences about places. Aiello et al. (2010) found that perceived quality of public spaces were positively associated with residential satisfaction. Nasar (1983) found that people preferred residential scenes that appeared well-maintained and clear to use. In Herzog’s (1992) study of urban spaces, coherence and complexity consistently predicted environmental preference. Nasar and Bokharaei (2017a, b) also found that bright lighting enhanced preferences of public spaces. In a virtual study, Nasar and Cubukcu (2011) found that preference of city environments increased as perceived mystery and surprise of those environments increased. It is worth mentioning that the association between environmental perception and preference extends to natural settings. Herzog and Leverich (2003) found that people liked forests that they perceived as legible and coherent. Chiang et al. (2014) found that people disliked forest trails for which they had situational concern about environmental threats. Another related line of research is that certain elements in public space can affect people’s experiences of public space. Abdulkarim and Nasar (2014a, b) demonstrated that public seating, sculpture, and food vendors made public plazas appear more visitable. Lindal and Hartig (2013) found that architectural variation, a concept similar to complexity, enhanced restorativeness. Other studies found that trees and vegetations enhanced the perceived restorativeness of public spaces (Lindal and Hartig, 2015; Rašković and Decker, 2015). Overall, there is a strong research foundation for linking environmental perception to environmental preference. In the current paper, we will examine the criterion validity of our scale by demonstrating how particular aspects of environmental perception can determine preferences about public spaces.

## Materials and Methods

We conducted a quantitative study to identify the core attributes underlying the environmental perception of public spaces. We first developed a typology of public spaces. We then constructed pictorial stimuli to represent this typology. Next, we administered an online survey through which research participants evaluated the pictorial stimuli of public spaces on the initial items we identified from the literature. Finally, we performed factor analyses to develop factor models of environmental perception of public spaces. Research-ethics approval was obtained prior to conducting the study.

### Stimuli

#### Typology of Public Spaces

We developed a typology of public spaces to operationalize public spaces. As Project for Public Spaces (2018) describes, public spaces are often “*used by many different people for many different purposes at many different times of the day and the year*” (p. 1). Public spaces is a broad concept; they are places that are open to all, allowing a vast variety of activities to take place. Public spaces serve a variety of functions and purposes, and can be categorized into different types (Carmona, 2010). Carr et al. (1992) categorized 11 major types of public space: *streets*, *squares and plazas*, *found/neighborhood spaces*, *public parks*, *greenways and parkways*, *memorials*, *markets*, *playgrounds*, *community open spaces*, *atriums/indoor marketplaces*, and *waterfronts*. Streets are pedestrian and vehicular corridors where people move on foot. Squares and plazas are multifunctional spaces available to all people. Found/neighborhood spaces are vacant or undeveloped spaces that are either ignored or not intended for a specific use. Public parks and greenways and parkways are green areas intended for social activities. Memorials memorialize people or important events. Markets are outdoor or exterior spaces used for shopping. Playgrounds are play areas that include play equipment (e.g., slides and swings). Community open spaces are spaces designed, developed, or managed by local residents on vacant land. Atriums/indoor marketplaces refer to indoor shopping areas. Waterfronts refer to open spaces along waterways in cities. Similarly, Gehl and Gemzøe (2001) identified five types of public space, among which four resemble Carr et al.’s (1992) categorization: *promenades* resembling streets, *main city squares* and *recreational squares* resembling squares and plazas, and *monumental squares* resembling memorials. Gehl and Gemzøe added *traffic squares* – public spaces for transport facilities such as transit stations or stops for subways or busses. Stanley et al. (2012) also identified six types of open space, among which five resemble the previous categorizations: *transport facilities*, *street*s, *plazas*, *parks and gardens*, and *incidental spaces* resembling Carr et al.’s (1992) found/neighborhood spaces. Stanley et al. (2012) added *recreational spaces* – specialized spaces designed or used for sports or exercises. Table 2 summarizes the different typologies of public spaces. Combining the overlapping types and retaining all that are unique, public spaces can be categorized into 12 unique types – *transport facility*, *street*, *square*, *recreational space*, *found neighborhood space*, *park*, *memorial*, *market*, *playground*, *community open space*, *indoor marketplace*, and *waterfront* – each serving a different function. We used this typology to operationalize public spaces in the current study.

**TABLE 2:** Typology of public spaces.

Unique Type	Carr et al., 1992	Gehl and Gemzøe, 2001	Stanley et al., 2012
Transport Facility		Traffic Squares	Transport Facilities
Street	Streets	Promenades	Streets
Square	Squares and Plazas	Main City Squares	Plazas
		Recreational Squares	
Recreational Space			Recreational Spaces
Found Neighborhood Space	Found/Neighborhood Spaces		Incidental Spaces
Park	Public Parks		Parks and Gardens
	Greenways and Parkways		
Memorial	Memorials	Monumental Squares	
Market	Markets		
Playground	Playgrounds		
Community Open Space	Community Open Spaces		
Indoor Marketplace	Atrium/Indoor Marketplace		
Waterfront	Waterfronts		

#### Pictorial Stimuli of Public Spaces

**FIGURE 1:**
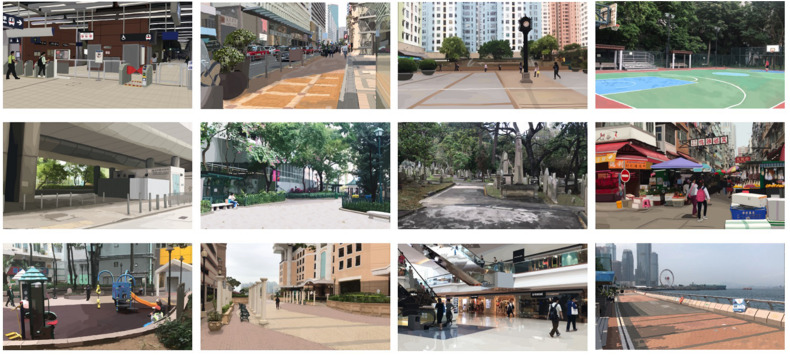
**(Top row)** From left to right, transport facility, street, square, and recreational space; **(middle row)** from left to right, found neighborhood space, park, memorial, and market; **(bottom row)** from left to right, playground, community open space, indoor marketplace, and waterfront.

We constructed a set of pictorial stimuli to represent the 12 types of public spaces. The use of static media to simulate environmental settings in research is supported by a meta-analysis by Stamps (2010), who showed that subjective evaluations of environmental settings on-site and their static simulations were very strongly correlated at *r* = 0.86. Based on our typology of public spaces, we started with 48 locations in Hong Kong (in which we are based) that fit the definitions of the 12 space types, four locations per type. We conducted three pilot studies online with a total of 310 local and non-local research participants to finalize the set of 12 public spaces to be used in the main study. Supplementary Appendix A reports the methods and results of the pilot studies. After confirming the 12 locations that would best represent the 12 types of public space, we visited those actual locations and took photographs of them, all using the same camera with the same focal length to ensure we had a consistent depth of field perspective looking into the locations. All photographs were taken during daytime on a weekend to control for the natural pedestrian flow. Using Adobe Photoshop, we then applied slight posterization to all photographs to give emphasis on the environmental settings over the realistic conditions of the actual locations. Figure 1 presents the final construction of the stimuli.

### Instruments

#### Initial Items

Our literature review identified 20 publications that operationalized environmental perception with self-report items. Those publications provided 210 measurement items in total, among which 47 items covered the 10 affective constructs and 163 items covered the 27 cognitive constructs. For parsimony, this item pool was reduced to just sufficiently cover all of the unique constructs in both domains. We carried out two procedures. First, we removed all duplicate items. For example, in the affective domain, we observed duplications of the item “*Boring/Uninteresting–Interesting*” among four studies (Nasar, 1983; Hanyu, 1997; Nasar and Cubukcu, 2011; Motoyama and Hanyu, 2014). Those four duplicate items were reduced to one item. Similarly in the cognitive domain, there was a duplication of a set of five “perceived crowding” items between two studies (Yüksel, 2009; Ho et al., 2020). Those two duplicate sets were reduced to one set. After removing duplicate items, we further reduced the number of items for constructs that were operationalized differently among studies. Between different operationalizations of the same construct, we opted for the one with fewest number of items. For example, two studies operationalized lighting in the cognitive domain. Herzog and Bryce (2007) used three items and van Rijswijk and Haans (2018) used six items. We kept Herzog and Bryce’s items but trimmed van Rijswijk and Haans’ items, as the former had fewer items. Following these procedures, 22 affective items and 63 cognitive items were retained in the item pool, as presented in Table 3.

**TABLE 3A:** Initial item pool (affective domain).

***Activity***
Dull–Lively
Inactive–Active
Sleepy–Arousing
***Aesthetics***
Ugly–Beautiful
Unaesthetic–Aesthetic
Unappealing–Appealing
Unattractive–Attractive
***Crime Rate***
High rate of robbery/burglary/assault–Low rate of robbery/burglary/assault
***Desirable Living Place***
Undesirable as a place to live–Desirable as a place to live
***Excitement***
Unstimulating–Stimulating
Boring–Exciting
***Interest***
Uninteresting–Interesting
***Pleasantness***
Uncomfortable–Comfortable
Unenjoyable–Enjoyable
Unpleasant–Pleasant
***Preference***
Bad–Good
Dislike–Like
Negative–Positive
Unfavorable–Favorable
***Relaxation***
Distressing–Relaxing
Upsetting–Calming
***Safeness***
Fearful–Safe

**TABLE 3B:** Initial item pool (cognitive domain 1 of 3).

***Age***
The elements in this place seem to be very old.
***Brightness***
This setting has very bright, clear lighting.
***Building Care***
The buildings in this place appear to be very well tended or well cared for.
***Clarity***
The use of this place and its parts are very ambiguous.
The use of this place and its parts are very clear.
***Coherence***
It is very easy to organize and structure the scene of this place.
The scene in this place hangs very well together.
***Complexity***
A great deal is going on in this place.
There is a lot to look at in this place.
This place contains many elements of different kinds.
***Composition***
It is very easy to structure and organize this place as a picture.
This place seems to be very well composed or well organized as a two-dimensional picture.
***Danger***
It is very likely I could be harmed in this place.
This place is very dangerous.
***Enclosure***
In this place I strongly feel being “inside looking out.”
This place gives me a strong feeling of being enclosed in a hiding place.
This place really makes me think I am viewing the environment from inside a hiding place.
***Landmarks***
This place contains distinctive or memorable objects or features that could serve as useful landmarks to help me find my way around.
***Legibility***
In this place it would be very easy to figure out where I am at any given moment.
In this place it would be very easy to find my way around.
In this place it would be very easy to find out my way back to any given point.

**TABLE 3C:** Initial item pool (cognitive domain 2 of 3).

***Lighting***
In this place the light level is high enough that I can see everything clearly.
The light in this place is very good.
To a large extent this place seems to be dominated by low light or deep shadow.
***Mystery***
To a large extent this place hides positive or negative encounters that might lie ahead.
To a large extent this place promises more to be seen if I could walk deeper in it.
***Naturalness***
There are many trees, vegetations, and flowers in this place.
***Nature Care***
The nature (foliage, vegetation) in this place appears to be very well tended or well cared for.
***Nuisance Elements***
Nuisances elements (e.g., wires, poles, fences, trash cans, signs, deterioration) are prominent in this place.
***Perceived Crowding***
This place is free for moving around.
This place is very cramped.
This place is very crowded.
This place is very spacious.
This place is very stuffy.
***Refuge***
To a large extent this place contains possible hiding places that I am viewing from the outside.
***Safety***
I have a very good overview over the environment of this place.
In this place, I can see objects very well.
In this place, I can see very well what is happening.
It is very easy for ill-intentioned people to find a hiding place in this place.
It is very easy to bring myself into safety in this place.
It would be very hard for an ill-intentioned person to entrap me in this place.
There are many areas in this place where a potential criminal can hide.
There is a large probability that an ill-intentioned person would hide in this place.
There is a large probability that I can escape this place in case of an emergency.

**TABLE 3D:** Initial item pool (cognitive domain 3 of 3).

***Situational Concern***
In this place, there is some wildlife that can harm people, such as snakes, bees, and toxic plants.
It is easy to get lost in this place.
There are potentially harmful animals and plants in this place.
There is possible danger from other people in this place.
This place is prone to crimes.
This place is prone to natural disasters such as storms, floods, and wildfires.
This place is unstable and unsafe.
This place is very dark, and it is not easy to find my way.
***Spaciousness***
I can see very deep and wide into this place from my point.
This place conveys a great deal of spaciousness or depth.
***Surprise***
In this place, there is a mismatch between what I expect to see and what I actually see.
***Typicality***
This place is a very good example of whatever category it belongs to.
This place seems to be a very representative example of its class.
***Uniform Lighting***
This place has uniform lighting.
***Upkeep***
This setting is very well upkept (well maintained).
***Vehicles***
Cars are prominent in this place.
***Visibility***
I can see very well all parts of this place without having my view blocked or interfered with.
It is very easy to move within or through this place.
It is very easy to see into this place.

#### Outcome Variables: Restorativeness, Safety, and Visitability

To examine the association between environmental perception of and preferences about public spaces, we included three outcome variables: perceived restorativeness, safety, and visitability. Measurement items for these variables were available from Abdulkarim and Nasar (2014b), Pals et al. (2014), and van Rijswijk and Haans (2018), respectively. Items are presented in Table 4. Perceived restorativeness refers to the extent to which a public space is perceived as relaxing and allowing its viewers to take a break from daily stressors. Perceived safety refers to the extent to which people feel safe and secure about a public space. Visitability examines the extent to which people perceive a public space as friendly for visit. These outcome variables will allow us to explore the impact of environmental perception of public spaces.

**TABLE 4:** Measurement items for outcome variables.

***Perceived Restorativeness***
In this place, I would be able to concentrate well.
In this place, I would be able to focus on myself.
In this place, I would be able to relax.
In this place, I would be able to release all tension.
In this place, my energy level would get renewed.
***Perceived Safety***
I feel very comfortable with the idea of having to walk into this place.
This place seems very safe.
To a large extent I would normally avoid a place like this during a nightly stroll.*
***Perceived Visitability***
I will stop at this place if I happen to be passing by.
I will walk out of my way to visit and spend time in this place.
I would regularly visit this place.
This is a place where I would choose to meet a friend.

*Reverse-coded item.

### Procedure

Data were collected through an online survey. Research participants were recruited on Amazon Mechanical Turk (MTurk). Collecting data on MTurk has the benefit of obtaining samples that are often more socio-economically and ethnically diverse than other forms of convenient samples (e.g., university psychology students; Berinsky et al., 2012; Casler et al., 2013). Each participant received USD$1.50 for participation and was randomly assigned to evaluating one of the 12 types of public space. The survey began with an introduction explicitly stating that the study was to understand the human experience of public spaces. After giving their informed consent, research participants were presented with the complete set of 12 images of public space; the order of the images being presented was random for every participant. After viewing the set, one of the 12 images was chosen randomly to be presented again; this time the presentation of the chosen image came with the definition of the corresponding space type. Presenting the image and the definition together was to ensure that the participants were conscious about which space type the given image was representing. The participants were then asked to evaluate their environmental experience of the public space as portrayed by the image; they were reminded to focus on the public space rather than the quality of the image, and that there were no right or wrong answers. The participants provided demographic information at the end of the survey.

Affective items were rated in a 7-point bipolar format. Cognitive items and items for outcome variables, plus 26 other items for another research purpose, were rated on a 7-point Likert scale (from *strongly disagree* to *strongly agree* coded from 1 to 7 with a midpoint *neither agree nor disagree* as 4). Bipolar items were always presented first. The order among the affective items and the order among the cognitive items were both randomized for every participant. Five instructed response items (IRIs) were included as attention check. IRI is an item to which there is an obvious and unambiguous, correct answer; it is widely used and acceptable in survey research (Gummer et al., 2018; Kam and Chan, 2018; Kung et al., 2018). In the current study, the IRI was: “*For this statement, please select [Strongly disagree/Disagree/Somewhat disagree/Neither agree nor disagree/Somewhat agree/Agree/Strongly agree].*” All five IRIs needed to be correctly answered for a participant’s responses to be considered as valid and included in the data analysis.

## Results

### Sample

A total of 1,892 responses were received. After excluding the responses that did not pass the attention check, a total of 1,650 cases were retained for the data analysis. The sample comprised 849 women and 793 men (8 preferred not to answer) whose average age was 37 years old (*SD* = 12.05; 32 preferred not to answer). Table 5 presents the sample’s demographics. Majority of the sample lived in North America (81.60%) and about a tenth in Asia (12.20%). Proportions were about the same between those who had attained a bachelor’s degree as their highest education level (41.90%) and those who had not (39.20%); the rest had attained an education level above a bachelor’s degree (18.50%). Most of the sample identified themselves as middle class (59.30%) and working class (31.70%). Table 6 presents the samples sizes of the 12 groups.^1^

**TABLE 5:** Demographics of sample.

Location	*n*	(%)
North America	1,347	(81.64)
South America	42	(2.55)
Africa	3	(0.18)
Europe	42	(2.55)
Asia	202	(12.24)
Australia/Oceania	4	(0.24)
Prefer not to answer	10	(0.61)

**Education**	***n***	**(%)**

Some high school	12	(0.73)
High school diploma or equivalent	134	(8.12)
Vocational training	30	(1.82)
Some college	321	(19.45)
Associate’s degree	150	(9.09)
Bachelor’s degree	691	(41.88)
Some post undergraduate work	41	(2.48)
Master’s degree	231	(14.00)
Specialist degree	2	(0.12)
Applied or professional doctorate degree	12	(0.73)
Doctorate degree	22	(1.33)
Prefer not to answer	4	(0.24)

**Class**	***n***	**(%)**

Poor	90	(5.45)
Working class	523	(31.70)
Middle class	979	(59.33)
Affluent	38	(2.30)
Prefer not to answer	20	(1.21)

**TABLE 6:** Sample sizes.

Public Space	*n* (%)	
Transport Facility	115	(6.97)
Street	134	(8.12)
Square	123	(7.45)
Recreational Space	115	(6.97)
Found Neighborhood Space	216	(13.09)
Park	112	(6.79)
Memorial	123	(7.45)
Market	121	(7.33)
Playground	122	(7.39)
Community Open Space	219	(13.27)
Indoor Marketplace	132	(8.00)
Waterfront	118	(7.15)

### Principal Component Analysis and Confirmatory Factor Analysis

We performed an exploratory factor analysis using principal component analysis (PCA) on a subset of the data and then cross-validated the results with a confirmatory factor analysis (CFA) on the remaining subset of the data, in order to develop factor models corresponding to the affective and cognitive domains respectively. The full sample was randomly split into two subsets by a 4:1 ratio. PCA was performed on the larger subset of 1,320 cases to develop the factor models; CFA was then performed on the smaller subset of 330 cases to assess the fit of the models resulted from the PCA. Eventually, the 22 affective items were reduced to 8 items capturing two factors; the 63 cognitive items were reduced to 22 items capturing six factors.

At the initial stage of each PCA, the factorability of the items was examined. We checked that all items correlated at least 0.30 with at least one other item, the diagonals of the anti-image correlation matrix were all above 0.50, and all item communalities were above 0.30. Next, we checked that Kaiser–Meyer–Olkin measure of sampling adequacy (KMO; Kaiser, 1970) was above 0.80 and Bartlett’s (1937) test of sphericity was significant. Parallel analysis (1,000 replications; Hayton et al., 2004) was used to estimate the number of factors to be retained in each model; component extraction with direct oblimin rotation (Δ = 0; Matsunaga, 2010) was performed. After rotation, we took note only of the items whose factor loading was greater than 0.49 and retained only the top four items for parsimony. We then gave each factor an interpretive label.

CFA was then performed using lavaan (Rosseel, 2012) with maximum likelihood estimation to test the factor models resulted from the PCA. To assess model fit, we referred to the Comparative Fit Index (CFI), the Tucker-Lewis Index (TLI), the Standardized Root Mean-Square Residual (SRMR), and the Root Mean-Square Error of Approximation (RMSEA). Whittaker’s (2016) recommended cutoffs are 0.90 or greater for CFI and TLI, 0.10 or less for SRMR, and 0.08 or less for RMSEA. We modified the models where appropriate and re-estimated them after modification.

#### Affective Domain

##### Principal component analysis

The dataset of 22 affective items was suitable for the analysis; KMO was 0.98 and Bartlett’s test of sphericity was statistically significant, χ^2^(231, *N* = 1,320) = 28,781.45, *p* < 0.001. Parallel analysis suggested that two factors should be retained, which explained 71.06% of the total variance. Factor loadings are presented in Table 7. Interpretive labels are suggested for the two factors: *comfort* and *activity*. Comfort describes if a public space conveys calming and relaxing feelings. Activity describes if the public space conveys arousing and lively feelings. The top four loading items were retained for each factor.

**TABLE 7:** Factor loadings in principal component analysis (affective domain).

Item	Comfort	Activity
Upsetting–Calming	0.893	–0.207
Distressing–Relaxing	0.886	–0.124
Uncomfortable–Comfortable	0.858	0.045
Fearful–Safe	0.816	–0.111
Unpleasant–Pleasant	0.809	0.146
Unfavorable–Favorable	0.775	0.217
Negative–Positive	0.771	0.211
Bad–Good	0.766	0.195
Dislike–Like	0.757	0.232
Undesirable place to live nearby–Desirable place to live nearby	0.743	0.079
Unattractive–Attractive	0.730	0.240
Unappealing–Appealing	0.725	0.286
Ugly–Beautiful	0.711	0.215
Unenjoyable–Enjoyable	0.701	0.298
High rate of robbery/burglary/assault–Low rate of robbery/burglary/assault	0.694	–0.233
Unaesthetic–Aesthetic	0.669	0.240

Inactive–Active	–0.047	0.839
Sleepy–Arousing	–0.031	0.775
Dull–Lively	0.164	0.775
Unstimulating–Stimulating	0.161	0.772
Boring–Exciting	0.189	0.750
Uninteresting–Interesting	0.432	0.537

##### Confirmatory factor analysis

The two-factor model, as resulted from the PCA, was estimated, χ^2^(19, *N* = 330) = 144.20, *p* < 0.001; CFI = 0.92; TLI = 0.89; SRMR = 0.07; RMSEA = 0.14 (90% CI: 0.12, 0.16), *p* < 0.001. At this point, the model satisfied the cutoffs of CFI and SRMR but not TLI and RMSEA. The modification index suggested a path to be added to allow covariance between two items – “*Upsetting–Calming*” and “*Distressing–Relaxing*”; that indicated the initial model might be inadequate in accounting for the relation between those two items. Those two items were both related to describing the comfort of a public space. Although they were already loaded onto the same factor, adding the suggested path should not affect the interpretation of the overall model. After adding the suggested path, the model was re-estimated, χ^2^(18, *N* = 330) = 88.27, *p* < 0.001; CFI = 0.96; TLI = 0.93; SRMR = 0.06; RMSEA = 0.11 (90% CI: 0.09, 0.13), *p* < 0.001. The model now satisfied the cutoffs of all fit indices except RMSEA; also, χ^2^ difference test indicated a significant improvement in model fit after model modification, χ^2^(1, *N* = 330) = 55.93, *p* < 0.001. Because *post hoc* model modification was performed, a correlation was calculated between the initial model parameter estimates and the parameter estimates from the modified (re-estimated) model, *r* = 0.86, *p* = 0.006; this indicated that parameter estimates were hardly changed despite modification of the model. The coefficients in both unstandardized and standardized forms are presented in Table 8.

**TABLE 8:** Factor loadings in confirmatory factor analysis (affective domain).

Item	*b*	*SE*	β
***Comfort***			
Upsetting–Calming	1.000		0.664
Distressing–Relaxing	1.193	0.075	0.730
Uncomfortable–Comfortable	1.462	0.116	0.912
Fearful–Safe	1.088	0.094	0.741
***Activity***			
Inactive–Active	1.000		0.800
Sleepy–Arousing	0.784	0.057	0.707
Dull–Lively	1.293	0.069	0.920
Unstimulating–Stimulating	1.030	0.061	0.834

**b*, unstandardized coefficient; *SE*, standard error; β, standardized coefficient; all *p*s < 0.001.*

#### Cognitive Domain

##### Principal component analysis

The dataset of 63 cognitive items was suitable for the analysis; KMO was 0.97 and Bartlett’s test of sphericity was statistically significant, χ^2^(1,953, *N* = 1,320) = 44,983.50, *p* < 0.001. Parallel analysis suggested that six factors should be retained, which explained 52.56% of the total variance. Factor loadings are presented in Table 9. Interpretive labels are suggested for the six factors: *legibility*, *enclosure*, *complexity*, *crime potential*, *wildlife*, and *lighting*. Legibility evaluates the extent to which a public space is easy to navigate within. Enclosure evaluates the extent to which the public space makes its viewers feel enclosed. Complexity refers to how much is going on in the public space. Crime potential refers to how much the public space is prone to crimes. Wildlife refers to the amount of trees, plants, and potential wildlife in the public space. Lighting describes the brightness and lighting quality in the public space. For lighting, only the top three loading items were retained because the fourth item had a loading below 0.49; for all the other factors, the top four loading items were retained.

**TABLE 9A:** Factor loadings in principal component analysis (cognitive domain 1 of 3).

Item	Legibility	Enclosure	Complexity	Crime Potential	Wildlife	Lighting
In this place it would be very easy to find out my way back to any given point.	0.752	0.029	0.030	–0.080	–0.006	–0.029
In this place it would be very easy to find my way around.	0.732	0.025	0.056	–0.081	–0.021	–0.108
In this place it would be very easy to figure out where I am at any given moment.	0.729	0.152	0.110	–0.119	0.013	–0.115
It is very easy to structure and organize this place as a picture.	0.612	–0.038	–0.185	0.024	0.001	–0.092
It is easy to get lost in this place.	–0.575	0.156	–0.403	0.244	–0.068	–0.126
It is very easy to organize and structure the scene of this place.	0.522	–0.047	–0.204	–0.027	0.015	–0.160
It is very easy to move within or through this place.	0.515	–0.250	–0.037	–0.003	–0.095	–0.252
It is very easy to see into this place.	0.492	–0.028	0.025	–0.062	–0.005	–0.382
In this place, I can see objects very well.	0.480	–0.090	–0.150	–0.023	0.053	–0.327
In this place, I can see very well what is happening.	0.474	–0.043	–0.078	–0.058	0.064	–0.359
This place seems to be very well composed or well organized as a two-dimensional picture.	0.466	–0.016	–0.305	–0.057	0.002	–0.053
There is a large probability that I can escape this place in case of an emergency.	0.390	–0.116	–0.021	–0.105	–0.178	–0.149
This place is free for moving around.	0.352	–0.325	–0.155	0.048	–.096	–0.291
I have a very good overview over the environment of this place.	0.348	0.012	–0.287	–0.169	–0.095	–0.173

This place is very stuffy.	–0.134	0.686	0.028	0.039	0.179	0.076
This place is very cramped.	–0.189	0.597	–0.116	0.089	0.171	0.241
In this place I strongly feel being “inside looking out.”	0.156	0.540	–0.081	0.028	–0.116	–0.021
This place gives me a strong feeling of being enclosed in a hiding place.	0.035	0.538	0.025	0.165	–0.154	0.255
This place really makes me think I am viewing the environment from inside a hiding place.	0.178	0.532	–0.005	0.078	–0.329	0.088
Cars are prominent in this place.	–0.044	0.530	–0.002	0.023	–0.058	–0.155
Nuisances elements (e.g., wires, poles, fences, trash cans, signs, deterioration) are prominent in this place.	0.040	0.502	0.018	0.241	0.112	0.052
This place is very crowded.	–0.286	0.491	–0.388	0.080	0.368	–0.062
This place is very dark, and it is not easy to find my way.	–0.199	0.348	0.091	0.224	–0.210	0.175
The use of this place and its parts are very ambiguous.	0.076	0.291	0.095	0.118	–0.259	–0.081

**TABLE 9B:** Factor loadings in principal component analysis (cognitive domain 2 of 3).

Item	Legibility	Enclosure	Complexity	Crime Potential	Wildlife	Lighting
There is a lot to look at in this place.	–0.194	0.001	–0.809	–0.047	–0.049	–0.104
To a large extent this place promises more to be seen if I could walk deeper in it.	–0.030	–0.066	–0.695	0.117	–0.077	–0.012
This place contains many elements of different kinds.	–0.072	0.064	–0.676	–0.019	–0.063	–0.067
A great deal is going on in this place.	–0.161	0.352	–0.621	–0.078	0.297	–0.177
This place seems to be a very representative example of its class.	0.283	–0.018	–0.500	–0.041	0.091	0.065
This place contains distinctive or memorable objects or features that could serve as useful landmarks to help me find my way around.	0.135	–0.025	–0.477	–0.084	–0.274	–0.017
The scene in this place hangs very well together.	0.308	0.017	–0.471	–0.163	–0.008	–0.079
This place is a very good example of whatever category it belongs to.	0.352	–0.159	–0.468	0.073	0.090	0.055
The use of this place and its parts are very clear.	0.350	0.066	–0.410	–0.050	0.166	0.018
This setting is very well upkept (well maintained).	0.193	–0.194	–0.305	–0.137	–0.003	–0.094
The buildings in this place appear to be very well tended or well cared for.	0.292	–0.128	–0.294	–0.120	0.002	–0.163

There are many areas in this place where a potential criminal can hide.	–0.043	–0.063	–0.079	0.797	0.015	0.076
This place is prone to crimes.	–0.087	0.037	0.118	0.778	0.116	–0.132
There is a large probability that an ill-intentioned person would hide in this place.	0.023	0.140	0.025	0.776	0.040	–0.013
There is possible danger from other people in this place.	–0.072	–0.043	0.021	0.774	0.085	–0.084
It is very easy for ill-intentioned people to find a hiding place in this place.	0.050	–0.022	–0.134	0.751	–0.060	0.184
It is very likely I could be harmed in this place.	0.032	0.160	0.119	0.717	0.037	–0.099
To a large extent this place hides positive or negative encounters that might lie ahead.	0.041	0.125	–0.160	0.596	–0.068	0.054
To a large extent this place contains possible hiding places that I am viewing from the outside.	0.133	0.064	–0.139	0.589	–0.152	0.269
This place is very dangerous.	–0.244	0.115	0.241	0.566	–0.032	–0.147
This place is unstable and unsafe.	–0.165	0.236	0.247	0.528	–0.053	–0.157
It would be very hard for an ill-intentioned person to entrap me in this place.	0.051	0.167	–0.075	–0.485	–0.160	–0.205
It is very easy to bring myself into safety in this place.	0.309	0.117	–0.291	–0.412	–0.118	–0.135

**TABLE 9C:** Factor loadings in principal component analysis (cognitive domain 3 of 3).

Item	Legibility	Enclosure	Complexity	Crime Potential	Wildlife	Lighting
There are many trees, vegetations, and flowers in this place.	0.035	–0.043	–0.089	–0.181	–0.758	0.116
In this place, there is some wildlife that can harm people, such as snakes, bees, and toxic plants.	–0.238	0.239	0.038	0.097	–0.601	–0.118
The nature (foliage, vegetation) in this place appears to be very well tended or well cared for.	0.140	–0.156	–0.178	–0.231	–0.562	0.030
There are potentially harmful animals and plants in this place.	–0.266	0.303	0.070	0.152	–0.499	–0.188
The elements in this place seem to be very old.	–0.169	0.215	0.035	0.128	–0.400	0.159
This place is prone to natural disasters such as storms, floods, and wildfires.	–0.083	0.143	0.041	0.241	–0.335	–0.253
In this place, there is a mismatch between what I expect to see and what I actually see.	–0.155	0.295	0.152	0.172	–0.296	–0.100

This place has uniform lighting.	0.139	0.135	–0.074	–0.113	0.109	–0.561
The light in this place is very good.	0.193	–0.021	–0.202	–0.188	0.029	–0.557
This setting has very bright, clear lighting.	0.170	0.035	–0.191	–0.184	0.104	–0.550
This place is very spacious.	0.231	–0.317	–0.098	0.109	–0.202	–0.489
In this place the light level is high enough that I can see everything clearly.	0.210	–0.079	–0.154	–0.188	0.060	–0.483
I can see very well all parts of this place without having my view blocked or interfered with.	0.339	0.036	0.044	–0.195	–0.057	–0.472
To a large extent this place seems to be dominated by low light or deep shadow.	0.047	0.367	0.024	0.224	–0.199	0.441
This place conveys a great deal of spaciousness or depth.	0.126	–0.237	–0.249	0.049	–0.207	–0.431
I can see very deep and wide into this place from my point.	0.360	0.019	–0.101	–0.042	–0.130	–0.419

##### Confirmatory factor analysis

The six-factor model, as resulted from the PCA, was estimated, χ^2^(215, *N* = 330) = 653.05, *p* < 0.001; CFI = 0.88; TLI = 0.85; SRMR = 0.10; RMSEA = 0.08 (90% CI: 0.07, 0.09), *p* < 0.001. At this point, the model satisfied the cutoffs of SRMR and RMSEA but not CFI and TLI. We observed a non-significant factor loading of one item – “*The nature (foliage, vegetation) in this place appears to be very well tended or well cared for*” (*p* = 0.291); that indicated the initial model might be inadequate in accounting for the variance of that item. That item focused on describing the maintenance of the nature of a public space, while the other three items loaded onto the same factor (wildlife) focused on describing the amount of nature in a public space. Excluding the maintenance item would allow the other amount items to convey a more focused meaning; in other words, dropping that item would improve the interpretation of the overall model. After dropping the non-significant item, the model was re-estimated, χ^2^(194, *N* = 330) = 484.07, *p* < 0.001; CFI = 0.91; TLI = 0.90; SRMR = 0.07; RMSEA = 0.07 (90% CI: 0.06, 0.08), *p* < 0.001. The model now satisfied the cutoffs of all fit indices. Because *post hoc* model modification was performed, a correlation was calculated between the initial model parameter estimates and the parameter estimates from the modified (re-estimated) model, *r* = 1.00, *p* < 0.001; this indicated that parameter estimates were almost unchanged despite modification of the model. The coefficients in both unstandardized and standardized forms are presented in Table 10.

**TABLE 10:** Factor loadings in confirmatory factor analysis (cognitive domain).

Item	*b*	*SE*	β
***Legibility***			
In this place it would be very easy to find out my way back to any given point.	1.000		0.876
In this place it would be very easy to find my way around.	0.912	0.054	0.774
In this place it would be very easy to figure out where I am at any given moment.	0.950	0.049	0.843
It is very easy to structure and organize this place as a picture.	0.831	0.054	0.731
***Enclosure***			
This place is very stuffy.	1.000		0.729
This place is very cramped.	1.206	0.103	0.790
In this place I strongly feel being “inside looking out.”	0.388	0.092	0.259
This place gives me a strong feeling of being enclosed in a hiding place.	0.874	0.097	0.564
***Complexity***			
There is a lot to look at in this place.	1.000		0.790
To a large extent this place promises more to be seen if I could walk deeper in it.	0.842	0.081	0.654
This place contains many elements of different kinds.	0.760	0.071	0.676
A great deal is going on in this place.	0.840	0.085	0.613
***Crime Potential***			
There are many areas in this place where a potential criminal can hide.	1.000		0.754
This place is prone to crimes.	1.031	0.068	0.846
There is a large probability that an ill-intentioned person would hide in this place.	1.035	0.071	0.808
There is possible danger from other people in this place.	0.916	0.070	0.735
***Wildlife***			
There are many trees, vegetations, and flowers in this place.	1.000		0.306
In this place, there is some wildlife that can harm people, such as snakes, bees, and toxic plants.	1.892	0.383	0.725
There are potentially harmful animals and plants in this place.	1.999	0.412	0.853
***Lighting***			
This place has uniform lighting.	1.000		0.640
The light in this place is very good.	1.202	0.097	0.863
This setting has very bright, clear lighting.	1.307	0.106	0.854

**b*, unstandardized coefficient; *SE*, standard error; β, standardized coefficient; all *p*s < 0.001.*

### Invariance Analysis

Noting that the online survey in the current study was written and administered entirely in English while we collected samples from locations where English might not necessarily be a primary language, we conducted an invariance analysis to assess the fit of our models across groups from different locations. We divided the entire sample into two groups. The first group comprised 1,351 participants who reported that they resided in North America or Australia/Oceania, where English should be their primary language (English primary group). The second group comprised 289 participants who reported that they resided in South America, Africa, Europe, or Asia, where English might not necessarily be their primary language (English non-primary group). We excluded 10 participants who did not provide their current location. CFAs were performed using lavaan (Rosseel, 2012) with maximum likelihood estimation to assess the fits of both the affective and cognitive models to both the English primary and non-primary groups. We used the same model-fit criteria as in the previous section.

The two-factor affective model fit both groups equally well [English primary group: χ^2^(18, *N* = 1,351) = 186.114, *p* < 0.001; CFI = 0.98; TLI = 0.96; SRMR = 0.05; RMSEA = 0.08 (90% CI: 0.07, 0.09), *p* < 0.001; English non-primary group: χ^2^(18, *N* = 289) = 45.306, *p* < 0.001; CFI = 0.97; TLI = 0.96; SRMR = 0.04; RMSEA = 0.07 (90% CI: 0.05, 0.10), *p* < 0.001]. The six-factor cognitive model also fit both groups equally well [English primary group: χ^2^(194, *N* = 1,351) = 1,339.192, *p* < 0.001; CFI = 0.92; TLI = 0.90; SRMR = 0.08; RMSEA = 0.07 (90% CI: 0.06, 0.07), *p* < 0.001; English non-primary group: χ^2^(194, *N* = 289) = 341.066, *p* < 0.001; CFI = 0.92; TLI = 0.91; SRMR = 0.06; RMSEA = 0.05 (90% CI: 0.04, 0.06), *p* < 0.001]. Factor loadings of both affective and cognitive models for both the English primary and non-primary groups are provided in Supplementary Appendix B. The factor loadings between the English primary and non-primary groups were significantly positively correlated in both the affective model (*r* = 0.92, *p* = 0.001) and the cognitive model (*r* = 0.80, *p* < 0.001). In sum, our invariance analysis found that the factor structures of both the affective and cognitive models fit equally well both groups and that factor loadings were similar across the two groups. These findings provide further support for the external validity of our results in the sense that they were replicable across sub-samples regardless of the differences in their primary languages.

### Composite Scores

Using simple unit weighting (i.e., averaging the item scores under the same factor), composite scores corresponding to the eight attributes of environmental perception (comfort, activity, legibility, enclosure, complexity, crime potential, wildlife, and lighting) and the three outcome variables (perceived restorativeness, safety, and visitability) were computed for each case in the sample. The correlations among the composite scores and the reliability of each score are presented in Table 11. The significant correlations ranged from 0.07 to 0.76, that is, weak to strong. Between environmental perception and the outcome variables, both restorativeness (*r* = 0.75, *p* < 0.001) and visitability (*r* = 0.68, *p* < 0.001) were most strongly positively correlated with comfort; safety was most strongly negatively correlated with crime potential (*r* = −0.71, *p* < 0.001). Among environmental perception, the significant correlations ranged from 0.07 to 0.64, that is, weak to moderate. Moderate correlations were found between comfort and legibility (*r* = 0.55, *p* < 0.001), activity and complexity (*r* = 0.64, *p* < 0.001), and legibility and lighting (*r* = 0.58, *p* < 0.001). Enclosure, crime potential, and wildlife correlated negatively with all the others. No significant correlations were found between comfort and wildlife (*p* = 0.126), activity and enclosure (*p* = 0.247), and enclosure and complexity (*p* = 0.765). Overall, Cronbach’s alphas ranged from 0.67 to 0.90, indicating acceptable reliability of the measurements. Mean composite scores are generated and reported in Table 12.

**TABLE 11:** Correlations and reliabilities.

		1	2	3	4	5	6	7	8	9	10	11
1	Comfort	*0.884*										
2	Activity	0.469*	*0.874*									
3	Legibility	0.552*	0.323*	*0.844*								
4	Enclosure	−0.254*	–0.028	−0.425*	*0.702*							
5	Complexity	0.352*	0.642*	0.236*	0.007	*0.771*						
6	Crime Potential	−0.494*	−0.220*	−0.484*	0.488*	−0.136*	*0.861*					
7	Wildlife	0.038	−0.111*	−0.116*	0.286*	−0.067*	0.204*	*0.668*				
8	Lighting	0.472*	0.402*	0.575*	−0.312*	0.361*	−0.455*	−0.097*	*0.823*			
9	Restorativeness	0.746*	0.383*	0.569*	−0.214*	0.364*	−0.481*	0.191*	0.506*	*0.898*		
10	Safety	0.658*	0.429*	0.603*	−0.416*	0.382*	−0.708*	−0.158*	0.565*	0.635*	*0.771*	
11	Visitability	0.682*	0.601*	0.556*	−0.209*	0.595*	−0.446*	–0.023	0.528*	0.756*	0.714*	*0.885*

Cronbach’s alphas are reported in italics along the diagonal; *p < 0.01.

**TABLE 12A:** Mean ratings of the 12 public spaces on environmental perception.

Public Space Type	Comfort	Activity	Legibility	Enclosure	Complexity	Crime Potential	Wildlife	Lighting
Transport Facility	4.34 (1.22)	4.72 (1.22)	4.96 (1.08)	3.26 (1.32)	4.60 (1.13)	4.15 (1.10)	1.73 (1.03)	5.17 (1.04)
Street	4.70 (0.99)	5.42 (0.99)	5.09 (0.99)	3.41 (1.09)	5.27 (1.05)	3.59 (1.14)	2.44 (1.33)	4.88 (1.26)
Square	5.75 (1.18)	5.32 (1.35)	5.77 (0.88)	2.69 (1.40)	4.64 (1.26)	2.88 (1.29)	3.21 (1.27)	5.47 (1.01)
Recreational Space	5.58 (1.08)	5.73 (1.02)	5.70 (0.72)	2.93 (1.01)	4.07 (1.14)	3.21 (1.11)	3.37 (1.13)	5.32 (0.80)
Found Neighborhood Space	3.75 (1.30)	2.82 (1.41)	4.25 (1.11)	3.73 (0.93)	3.01 (1.31)	5.03 (0.98)	2.93 (1.19)	3.38 (1.47)
Park	6.12 (0.75)	5.00 (1.04)	5.39 (0.87)	3.06 (1.04)	4.69 (0.96)	3.13 (1.18)	3.87 (0.82)	5.09 (0.89)
Memorial	4.66 (1.41)	3.56 (1.04)	4.27 (1.24)	3.47 (1.10)	4.29 (1.03)	3.84 (1.40)	3.89 (1.02)	3.77 (1.19)
Market	4.79 (1.20)	6.00 (0.88)	4.39 (1.23)	4.19 (0.96)	5.55 (0.98)	4.04 (1.37)	2.66 (1.36)	4.09 (1.19)
Playground	5.58 (1.02)	5.63 (0.93)	5.58 (0.89)	2.96 (1.18)	4.63 (0.92)	2.84 (1.09)	3.04 (1.00)	5.19 (1.08)
Community Open Space	6.03 (0.81)	4.47 (1.00)	5.60 (0.89)	2.61 (1.17)	4.33 (1.02)	2.56 (1.11)	2.54 (1.12)	5.55 (0.94)
Indoor Marketplace	5.37 (0.97)	5.59 (1.06)	5.21 (0.97)	3.10 (1.32)	5.46 (0.87)	3.29 (1.34)	1.76 (1.03)	5.60 (0.98)
Waterfront	5.79 (0.98)	5.45 (1.03)	5.67 (0.85)	2.66 (1.20)	5.07 (1.07)	3.23 (1.24)	2.30 (1.31)	5.32 (1.17)

*All ratings were measured on a 7-point scale with 4 being the mid-point; standard deviations are in brackets.*

**TABLE 12B:** Mean ratings of the 12 public spaces on outcome variables.

Public Space Type	Restorativeness	Safety	Visitability
Transport Facility	3.38 (1.29)	4.18 (1.21)	3.59 (1.22)
Street	3.92 (1.29)	4.92 (1.01)	4.63 (1.25)
Square	5.19 (1.20)	5.39 (1.10)	5.23 (1.34)
Recreational Space	4.98 (1.04)	4.89 (1.09)	4.84 (1.10)
Found Neighborhood Space	3.19 (1.44)	3.08 (1.37)	2.76 (1.42)
Park	5.53 (0.95)	5.27 (0.99)	5.32 (0.99)
Memorial	4.31 (1.41)	4.01 (1.42)	3.58 (1.53)
Market	4.02 (1.31)	4.61 (1.19)	5.12 (1.01)
Playground	4.62 (1.16)	5.21 (0.94)	4.92 (1.10)
Community Open Space	5.28 (0.91)	5.64 (1.05)	5.36 (0.92)
Indoor Marketplace	4.41 (1.15)	5.42 (1.02)	5.14 (1.26)
Waterfront	5.47 (0.98)	5.28 (1.09)	5.64 (1.03)

*All ratings were measured on a 7-point scale with 4 being the mid-point; standard deviations are in brackets.*

### Differentiating the 12 Public Spaces by Environmental Perception

Attributes of environmental perception would not be practical if they could not differentiate among different environmental settings. We therefore evaluated the extent to which the attributes of environmental perception could indeed differentiate among the 12 public-space images. We compared every possible pairs of public-space images on each of the eight attributes of environmental perception (comfort, activity, legibility, enclosure, complexity, crime potential, wildlife, and lighting) in terms of statistical and practical significance.^2^

There was a fair amount of variation among the 12 spaces on all attributes (see Table 12). A one-way multivariate analysis of variance (MANOVA) comparing the 12 spaces on the eight attributes found a statistically significant difference, *F*(88,4,468.52) = 16.29, *p* < 0.001; Wilk’s Λ = 0.16, $\eta_{\text{p}}^{2}$ = 0.20. Eight one-way ANOVAs then tested differences among the 12 spaces on each attribute separately. Since there were eight comparisons (i.e., eight attributes), the alpha level was adjusted to 0.05/8 = 0.00625 using a Bonferroni correction. There were still statistically significant differences in all attributes among the 12 public-space images, in terms of comfort [*F*(11,687) = 25.69, *p* < 0.001, $\eta_{\text{p}}^{2}$ = 0.29], activity [*F*(11,687) = 43.10, *p* < 0.001, $\eta_{\text{p}}^{2}$ = 0.41], legibility [*F*(11,687) = 19.14, *p* < 0.001, $\eta_{\text{p}}^{2}$ = 0.24], enclosure [*F*(11,687) = 9.58, *p* < 0.001, $\eta_{\text{p}}^{2}$ = 0.13], complexity [*F*(11,687) = 24.11, *p* < 0.001, $\eta_{\text{p}}^{2}$ = 0.28], crime potential [*F*(11,687) = 18.11, *p* < 0.001, $\eta_{\text{p}}^{2}$ = 0.23], wildlife [*F*(11,687) = 22.35, *p* < 0.001, $\eta_{\text{p}}^{2}$ = 0.26], and lighting [*F*(11,687) = 25.40, *p* < 0.001, $\eta_{\text{p}}^{2}$ = 0.29].

How the 12 public spaces were different from each other on each attribute was examined by a series of Tukey’s HSD post-hoc tests. Detailed results are reported in Supplementary Appendix C. Given the average group sample size of approximately 50 resulting in large statistical power to detect statistical significance, we also considered if the mean differences (as measured on a 7-point scale) were greater than ±1 as an indication of practical significance. Table 13 is a frequency table that summarizes the number of times a public-space image was statistically and practically different from another image on a given attribute. For example, transport facility was statistically and practically different from seven other space types in comfort, but it was not statistically nor practically different from the others in legibility and enclosure. The bottom row shows the total number of times the 12 spaces differed from each other on a given attribute. A larger number indicates that the 12 public spaces are more likely to differ from each other on the corresponding attribute. Comfort, activity, and lighting are the best three attributes in differentiating the 12 spaces, whereas enclosure is the least discriminating attribute. Overall, these results show that, to various extents, the eight attributes of environmental perception are all able to differentiate among different types of public spaces, which demonstrate the practical utility of the scale of environmental perception.

**TABLE 13:** Frequencies of statistically and practically significant differences among the 12 public spaces on environmental perception.

Public Space Type	Comfort	Activity	Legibility	Enclosure	Complexity	Crime Potential	Wildlife	Lighting	Total
Transport Facility	7	4	0	0	1	4	6	3	25
Street	4	2	0	0	2	2	2	2	14
Square	4	2	3	2	1	3	2	3	20
Recreational Space	2	4	3	1	4	1	3	3	21
Found Neighborhood Space	8	10	6	3	11	9	2	9	58
Park	5	3	2	1	1	2	6	2	22
Memorial	4	9	6	0	3	3	6	9	40
Market	3	5	5	7	4	3	2	7	36
Playground	2	3	3	1	1	4	2	3	19
Community Open Space	5	5	3	2	3	5	2	3	28
Indoor Marketplace	2	3	0	1	4	1	6	3	20
Waterfront	4	2	3	2	1	1	3	3	19
Total	50	52	34	20	36	38	42	50	322

*The mean difference between two space types on a given attribute was considered statistically significant if the corresponding *p-*value as derived from the Tukey’s HSD test was below 0.00625, as adjusted with a Bonferroni correction of *p* = 0.05/8. The mean difference between two space types on a given attribute was considered practically significant if its absolute value was greater than 1, given that all ratings were measured on a 7-point scale.*

### Multiple Regression of Outcome Variables on Environmental Perception

Multiple regressions were performed to examine the criterion validity of the scale of environmental perception in predicting evaluation of public spaces, i.e., perceived restorativeness, safety, and visitability. Noting the moderate-to-strong correlations among the variables, we observed variance inflation factors (VIFs) to detect potential threat of multicollinearity. All VIFs were below the threshold of 10 as recommended by Myers (1990; also see O’Brien, 2007), which indicated there was a negligible impact of multicollinearity. Results of the regressions are organized in Table 14, a summary is also provided below.

**TABLE 14:** Multiple regression of outcome variables on environmental perception.

	*b*	*SE*	β	*t*	*p*	Partial Correlation	VIF
**DV: Restorativeness**							
Comfort	0.539	0.022	0.489	24.580	0.000	0.519	1.954
Activity	–0.038	0.021	–0.037	–1.834	0.067	–0.045	2.010
Legibility	0.240	0.025	0.190	9.562	0.000	0.230	1.944
Enclosure	0.034	0.021	0.028	1.595	0.111	0.039	1.533
Complexity	0.150	0.022	0.131	6.919	0.000	0.168	1.764
Crime Potential	–0.161	0.019	–0.157	–8.294	0.000	–0.201	1.762
Wildlife	0.255	0.017	0.233	15.114	0.000	0.350	1.170
Lighting	0.100	0.020	0.094	4.931	0.000	0.121	1.775
**DV: Safety**							
Comfort	0.263	0.020	0.252	13.173	0.000	0.309	1.954
Activity	0.042	0.019	0.043	2.219	0.027	0.055	2.010
Legibility	0.164	0.023	0.137	7.188	0.000	0.175	1.944
Enclosure	–0.062	0.019	–0.055	–3.233	0.001	–0.080	1.533
Complexity	0.155	0.020	0.142	7.841	0.000	0.190	1.764
Crime Potential	–0.404	0.018	–0.416	–22.897	0.000	–0.492	1.762
Wildlife	–0.029	0.015	–0.028	–1.892	0.059	–0.047	1.170
Lighting	0.091	0.019	0.089	4.906	0.000	0.120	1.775
**DV: Visitability**							
Comfort	0.373	0.023	0.320	16.183	0.000	0.371	1.954
Activity	0.167	0.022	0.152	7.585	0.000	0.184	2.010
Legibility	0.240	0.026	0.179	9.093	0.000	0.219	1.944
Enclosure	0.012	0.022	0.010	0.561	0.575	0.014	1.533
Complexity	0.377	0.023	0.310	16.498	0.000	0.377	1.764
Crime Potential	–0.125	0.020	–0.115	–6.133	0.000	–0.150	1.762
Wildlife	0.058	0.018	0.050	3.259	0.001	0.080	1.170
Lighting	0.063	0.021	0.056	2.956	0.003	0.073	1.775

**b*, unstandardized coefficient; *SE*, standard error; β, standardized coefficient; *t*, t statistic; *p*, *p-*value; VIF, variation inflation factor.*

Environmental perception significantly predicted perceived restorativeness, *F*(8,1,641) = 411.732, *p* < 0.001, *R*^2^ = 0.667. All variables except activity and enclosure contributed statistical significance to the prediction. Higher perceived restorativeness could be predicted by higher comfort, higher legibility, higher complexity, lower crime potential, higher wildlife, and higher lighting (*p*s < 0.05).

Environmental perception significantly predicted perceived safety, *F*(8,1,641) = 463.331, *p* < 0.001, *R*^2^ = 0.693. All variables except wildlife contributed statistical significance to the prediction. Higher perceived safety could be predicted by higher comfort, higher activity, higher legibility, lower enclosure, higher complexity, lower crime potential, and higher lighting (*p*s < 0.05).

Environmental perception significantly predicted perceived visitability, *F*(8,1,641) = 419.523, *p* < 0.001, *R*^2^ = 0.672. All variables except enclosure contributed statistically significance to the prediction. Higher perceived visitability could be predicted by higher comfort, higher activity, higher legibility, higher complexity, lower crime potential, higher wildlife, and higher lighting (*p*s < 0.05).

Overall, these results demonstrate that our scale could predict perceived restorativeness, safety, and visitability about public spaces, and thereby provide support for the criterion validity of the scale.

## Discussion

### Major Findings

The current study addresses the need for a comprehensive tool for measuring subjectively perceived quality of public spaces. Through PCAs and CFAs, we developed factor models that consist of two affective factors (comfort and activity) and six cognitive factors (legibility, enclosure, complexity, crime potential, wildlife, and lighting). The corresponding scale items should sufficiently capture the core attributes underlying environmental perception of public spaces. Our scale allows us to compare different types of public spaces along the same standardized terms. With this new scale, we are able to describe, in a common language, the similarities as well as differences among different public spaces. Furthermore, this scale enables us to measure perception of public spaces on a quantitative basis, facilitating incorporation of subjective perception of public spaces into other research frameworks such as quality of life (Pacione, 2003). Overall, this scale should open new possibilities in research where public spaces is concerned.

Our factor structures of perception of public spaces follow the affective-cognitive distinction, which is prominent in environmental-perception research. By consolidating constructs and items from previous studies, our scale presents a succinct model of environmental perception. Initially there were 47 items measuring 10 unique affective constructs and 163 items measuring 27 unique cognitive constructs. The final affective model retained 8 affective items originated from 5 affective constructs that are now grouped into two factors. Comfort covers pleasantness, relaxation, and safeness. Activity covers activity and excitement. The final cognitive model retained 22 cognitive items originated from 12 cognitive constructs that are now grouped into six factors. Legibility covers legibility and composition. Enclosure covers enclosure and perceived crowding. Complexity covers complexity and mystery. Crime potential covers safety and situational concern. Wildlife covers naturalness and situational concern. Lighting covers lighting, brightness, and uniform lighting. We do not intend to claim that these 17 constructs in our model are sufficient in mapping the full spectrum of environmental perception of public spaces. We do hope to claim, however, those 17 constructs are necessary in describing the common experiences of public spaces. Our model may be further developed by other researchers through adding extra constructs or refining an existing construct depending on the specific research contexts.

Our scale could predict preferences about public spaces, and that re-confirms our knowledge about the association between environmental perception and preference. Perceived restorativeness of public spaces was predicted by higher comfort, higher legibility, higher complexity, lower crime potential, higher wildlife, and higher lighting. Perceived safety was predicted by higher comfort, higher activity, higher legibility, lower enclosure, higher complexity, lower crime potential, and higher lighting. Visitability was predicted by higher comfort, higher activity, higher legibility, higher complexity, lower crime potential, higher wildlife, and higher lighting. Not only do these results confirm the findings of previous research, but they also highlight the importance of the various aspects of environmental perception. At the practical level, for example, urban planners could use our model and scale to examine which aspects of public-space perception may account for other urban-related concepts such as inhabitants’ perceived insecurity, residential satisfaction, etc. At the theoretical level, our findings contribute to establishing the link between perceived attributes of an environment and preference of that environment.

### Limitations

Our findings are limited by the use of pictorial stimuli to represent environmental settings of public spaces. There is an obvious difference between experiencing a real-world environment and a simulated environment. Being situated in a real-world environment, a person receives visual, audio, olfactory, and tactile sensations whereas our stimuli were only static, two-dimensional visual stimuli. Also, a real-world environment provides a 360°, immersive experience with the environment whereas our pictorial depiction provided only a restricted point of view into the environment. That being said, the validity of using pictorial stimuli to simulate public spaces is supported by a meta-analysis of 17 empirical studies that evaluated environmental settings either on-site or through static simulations (Stamps, 2010); subjective evaluations of environmental settings on-site and their static simulations were very strongly correlated at *r* = 0.86. Thus, we are confident that our static pictorial stimuli were adequate in representing the actual locations in the real world. Also, using pictorial stimuli provided us with stronger experimental control over the research participants’ experiences. By adopting a single visual point of view into the public spaces in the current study, we could standardize the experiences of public spaces among all research participants. To overcome the limitation of the use of static stimuli, we suggest future studies to examine perception of public spaces in the real world or through a medium that allows a more immersive environmental experience. Higher-fidelity simulations such as videos and virtual reality, and stimuli accompanied by audios are good examples. Field experiments, while affording much less experimental control, should also be considered for maximizing the ecological validity of research findings.

The current findings are also limited by the use of only 12 images built upon a self-developed typology to represent the broad concept of public spaces. In reality, there are endless possibilities in how public spaces take shape across cultures and histories. It is impossible to exhaust all environmental settings that meet the definition of public spaces in one single study. Knowing the challenges in representing the vast notion of public spaces, we were cautious in developing the theoretical typology of public spaces and identifying the most appropriate images to our knowledge to depict that typology. Our typology is a result of combining well-established typologies in the literature; those typologies categorize public spaces according to their functions and purposes. Our pilot studies evaluated real-world public spaces in terms of their fit in representing our typology; our participant samples included both people local and foreign to the geographical region from which our public spaces were selected. In other words, the 12 public-space images of the current study had a theoretical root and were constructed with respect for real populations from different cultural contexts. Thus, although our collection of public spaces were limited, they should represent the most common types of public spaces. Future studies that cross-validate our scale should consider examining public spaces that are atypical or do not fit easily into our typology. Evaluating atypical public spaces can help uncover additional components or sub-components underlying environmental perception of public spaces.

The current findings are based on a convenient sample recruited on Amazon MTurk. As the individuals who make themselves available on MTurk are supposedly motivated by monetary rewards, some might question if such an unsupervised sample would show a genuine concern and interest for the welfare of scientific research. Research has shown that MTurk samples respond in a manner consistent with other convenient samples to experimental stimuli in framing experiments (Berinsky et al., 2012) and behavioral experiments (Casler et al., 2013), and thereby lends support to the reliability of the data collected through MTurk. We also included attention check in our survey as often suggested (Goodman et al., 2013; Kees et al., 2017). While correctly answering all attention-check items could not prove that a research participant had paid full attention throughout the survey, we excluded participants who failed attention check to safeguard the quality of the data at some level. Thus, despite the unsupervised nature of the MTurk samples, data-quality research findings and the attention-check mechanisms we employed provide us with the confidence about the reliability of our data. Future studies of public-space perception should incorporate alternative data sources. For example, respondents in field study are “real” people who might not necessarily behave the same way as people responding to an online survey. Actual field data will provide a good opportunity to cross-validate the scale.

Finally, there is the related issue regarding the cross-cultural nature of our online survey. Smith (2004) discusses the challenges of conducting research at the cross-cultural level. One major challenge relates to the language or linguistic aspects of the research materials. For example, questionnaire items might no longer convey the same meanings after being translated to a different language. Or some concepts simply do not exist in some cultures. Furthermore, some research procedures might not make sense to people from a culture different from that of the researcher. Majority of our sample resided in North America, while others resided in South America, Africa, Europe, Asia, and Australia/Oceania (see Table 5). Since our online survey was constructed and administered in English entirely, people outside the North America, where English might not necessarily be their primary language, could have responded differently to our survey due to linguistic differences. As our analysis has shown, our factor structures could fit both the English primary and non-primary groups equally well; that gives us confidence about our findings. Certainly, there was only a small proportion of samples that were recruited from outside the North America. Thus, further studies may try to establish proper cross-cultural comparisons. For example, data collection may be restricted to a particular location in the world. That way, we could systematically manipulate the cultural context where we collect our samples.

## Data Availability Statement

The raw data supporting the conclusions of this article will be made available by the authors, without undue reservation.

## Ethics Statement

The studies involving human participants were reviewed and approved by The Survey and Behavioural Research Ethics Committee of The Chinese University of Hong Kong. The patients/participants provided their written informed consent to participate in this study.

## Author Contributions

RH was the principal investigator of this research and was responsible for creating the main content of this manuscript. WTA was a co-principal investigator and was involved in the design, methodology, analysis, and reporting of this manuscript. Both authors contributed to the article and approved the submitted version.

## Conflict of Interest

The authors declare that the research was conducted in the absence of any commercial or financial relationships that could be construed as a potential conflict of interest.

## Footnotes

1 To be precise, there were 56 groups of research participants. The current study was part of a larger research project, which was also interested in the effects of *mislabeling*, *prompting*, and *street performance* (i.e., the act of performing or entertaining in public spaces with the intention of seeking donations from passersby). To examine the effect of mislabeling, two extra space types – a mislabeled found neighborhood space and a mislabeled community open space – were added to the research design. When presented with their assigned public-space image, the participants in the Mislabel conditions were provided with a space-type definition that was deliberately mislabeled; found neighborhood space was mislabeled as street and community open space was mislabeled as square. To examine the effect of prompting, the 14 space types (12 originals plus 2 mislabels) were doubled by the variation between prompting and no prompting. Participants in the Prompt conditions were instructed to actively imagine themselves using the space on a regular basis, whereas those in the No-Prompt conditions received no such instructions. To examine the effect of street performance, we manipulated all images between with and without an animated event of street performance. Participants in the No-Street-Performance conditions evaluated the public-space images as they were originally constructed whereas those in the Street-Performance conditions evaluated the exact same images but with an addition of a street performer playing a guitar and interacting with two adult passersby. In sum, the original 12 public-space types plus 2 extra mislabeled versions, doubled by the variation of prompting, and then doubled by the addition of street performance, resulted in a total of (12 + 2) × 2 × 2 = 56 groups of research participants in the current study.

2 In this analysis, we excluded the samples of the Mislabel groups and the Street-Performance groups (see description in Footnote 1), given the fundamental interest here was to differentiate the 12 public spaces as in their base (unmanipulated) settings.
