# Supplementary material for: Scale Development for Environmental Perception of Public Space
Source: Front Psychol. 2020 Nov 23;11:596790. doi: 10.3389/fpsyg.2020.596790 (PMC7719834; doi:10.3389/fpsyg.2020.596790)
Supplement: Supplementary file 1 [file Table_1.DOCX]

Appendix A

**Pilot Studies: Construction of Pictorial Stimuli of Public Spaces**

Based on our typology of public spaces, we identified 48 locations in Hong Kong that fit the definitions of the 12 space types. Using Google Street View, we captured an image of each location. We then conducted a series of recognition tasks to evaluate those 48 images (locations) and determine those that best represented the 12 space types. Through an online survey, we recruited local and non-local samples to ensure that our final results would be relevant to both groups of individuals familiar and unfamiliar with the selected locations. Local participants were undergraduate students taking a general-psychology course; they received course credit for participation. Non-local participants were workers on Amazon Mechanical Turk who had never visited Hong Kong; they received US$0.5 per person for participation. In total, three rounds of data collection and analysis were conducted.

**Pilot Study 1**

The local sample comprised 71 women and 28 men whose average age was 19.13 years (*SD* = 1.42); the non-local sample comprised 37 women and 65 men whose average age was 29.17 years (*SD* = 5.82). After giving their informed consent, the participants were introduced to the labels of the 12 space types (e.g., “Playground”) in a random order. They were then presented with 12 images, also in a random order; each was a location intended to represent one of the 12 space types. For each space type, only one image (out of four) was randomly chosen and presented. In other words, each participant was presented with a completely different and random set of images (locations). For each image, the participants were provided with the exact same 12 space-type labels that were introduced to them earlier. Their task was to select the space type(s) that they thought was a suitable descriptor of the given image. For any given image, the participants were allowed to select as many space types as they saw fit; thus, it was possible they would select none or all of the space types.

Whether a given image and a space type were statistically significantly associated was evaluated using a binominal test: Given that the random chance of a space type being selected as a suitable descriptor for an image is 50%, an image (location) would be considered as an appropriate representation of its intended space type if it was recognized beyond chance at the Type I error rate of .05 by both the local and non-local samples as the intended space type and the intended space type only.

For six space types – transport facility, street, park, memorial, market, and waterfront – we could identify at least one image (location) that satisfied the said criterion. For the other six space types, we suspected that the results were influenced by the difference in the usage of the space-type labels between the local and non-local samples. Such suspicion was best manifested in the images regarding recreational space and playground. The images intended to represent recreational space were all most recognized by the local sample as recreational space (i.e., “correct” response) but were all most recognized by the non-local sample as playground (i.e., “incorrect” response). Conversely, the images intended to represent playground were all most recognized by the local sample as playground (i.e., “correct” response) but were all most recognized by the non-local sample as recreational space (i.e., “incorrect” response). In both cases, the recognition rates of the successful labels were all beyond 95% chance level while those of the unsuccessful labels were not. In other words, in at least the cases regarding recreational space and playground, the images (locations) were fairly distinct in terms of their perceived space type, but at the same time those images (locations) seemed to be mapped in consistently opposite directions between the local and non-local samples. This led us to suspect there was a consistent difference in the usage of certain space-type labels between the two samples. To address the ambiguity in the space-type labels, we conducted Pilot Study 2 with a new, independent non-local sample, and we provided this new sample with not only the labels of the space types (e.g., “Playground”), but also the complete definitions (e.g., “Play area that includes play equipment such as slides and swings”) that would more accurately capture the features of the corresponding space type.

**Pilot Study 2**

The second non-local sample comprised 16 women and 36 men whose average age was 32.17 years (*SD* = 8.32). Procedures were similar to Pilot Study 1. After giving their informed consent, the participants were introduced to the labels *and* definitions of the 12 space types in a random order. They were then presented with 12 images, also in a random order; each was a location intended to represent one of the six space types whose results were inconclusive in Pilot Study 1 (i.e., square, recreational space, found neighborhood space, playground, community open space, and indoor marketplace). For each space type, only two images (out of four) were randomly chosen and presented. In other words, each participant was presented with a completely different and random set of images (locations). For each image, the participants were provided with the exact same 12 space-type labels *and* definitions that were introduced to them earlier. Their task was to select the space type(s) that they thought was a suitable descriptor of the given image. For any given image, the participants were allowed to select as many space types as they saw fit; thus, it was possible they would select none or all of the space types.

Using the same criterion as Pilot Study 1, we examined the local-sample data from Pilot Study 1 and the non-local-sample data from Pilot Study 2. For three space types – recreational space, playground, and indoor marketplace – we could identify at least one image (location) that satisfied the said criterion. Regarding square, one of the images (locations) was given extra consideration: Although the rate of it being recognized as square was beyond chance at the Type I error rate of .05 in the local sample, it only achieved a Type I error rate of .08 in the non-local sample (it would have satisfied the criterion if one more participant had selected it as a square). However, when the analysis was done with the local and non-local samples combined, the image satisfied the criterion. Thus, we decided to accept that image as an appropriate representation of square.

**Pilot Study 3**

Pilot Studies 1 and 2 helped to determined usable images (locations) that would reasonably represent 10 of the 12 space types, but results remained inconclusive regarding two space types, found neighborhood space and community open space. We were uncertain if the images (locations) we had initially sourced really had potential for representing those two specific space types, so we consulted an academic expert in urban studies in Hong Kong and 10 additional images (locations) were suggested to us, five per type. We conducted Pilot Study 3 to test those 10 images with a new, independent local sample and a new, independent non-local sample.

The second local sample comprised 19 women and 9 men whose average age was 18.93 years (*SD* = 1.02); the third non-local sample comprised 18 women and 11 men whose average age was 31.12 years (*SD* = 6.78). Procedures were similar to the previous pilot studies. After giving their informed consent, the participants were introduced to the labels and definitions of the 12 space types in a random order. They were then presented with 10 images, also in a random order; half were images intended to represent found neighborhood space and the other half community open space. For each image, the participants were provided with the exact same 12 space-type labels and definitions that were introduced to them earlier. Their task was to select the space type(s) that they thought was a suitable descriptor of the given image. For any given image, the participants were allowed to select as many space types as they saw fit; thus, it was possible they would select none or all of the space types.

Using the same criterion as the previous pilot studies, we examined the data provided by the new local and non-local samples. None of the 10 images satisfied the said criterion. Put together the results across the three pilot studies, it seemed rather difficult to determine an image (location) that would unambiguously represent found neighborhood space and community open space in both the local and non-local eyes. That remained true even after we consulted an expert in the public spaces of Hong Kong. One possible explanation is that the defining features of those two space types tend not to be observable through pictorial stimuli. Recall that found neighborhood space is defined as “*vacant or undeveloped space that is ignored or not intended for a specific use*” and that community open space is “*designed, developed, or managed by local residents on vacant land*”. These defining features might not be easily observable through pictorial stimuli. We acknowledge the limitations of using pictorial stimuli to represent the full spectrum of public spaces. At this point we relaxed the criterion for determining the representative images (locations) for found neighborhood space and community open space. With the local and non-local samples combined, we considered the results of all 18 images (i.e., 8 we had initially sourced and 10 we later obtained from the urban-studies expert). We chose the images (locations) that were recognized by the greatest proportion of the samples as the intended space type.
