# Supplementary material for: Scale Development for Environmental Perception of Public Space
Source: Front Psychol. 2020 Nov 23;11:596790. doi: 10.3389/fpsyg.2020.596790 (PMC7719834; doi:10.3389/fpsyg.2020.596790)
Supplement: Supplementary file 2 [file Table_2.docx]

Appendix B

**Factor Loadings of Affective and Cognitive Perception of Public Spaces across**

**English Primary and Non-primary Groups**

*Factor Loadings of Affective Model for English Primary Group*

| Item | *b* | *SE* | β |
| --- | --- | --- | --- |
| *Comfort* |  |  |  |
| Upsetting–Calming | 1.000 |  | 0.721 |
| Distressing–Relaxing | 1.148 | 0.031 | 0.770 |
| Uncomfortable–Comfortable | 1.409 | 0.048 | 0.928 |
| Fearful–Safe | 1.046 | 0.040 | 0.749 |
| *Activity* |  |  |  |
| Inactive–Active | 1.000 |  | 0.780 |
| Sleepy–Arousing | 0.863 | 0.031 | 0.724 |
| Dull–Lively | 1.247 | 0.036 | 0.886 |
| Unstimulating–Stimulating | 1.105 | 0.033 | 0.846 |

*Note*. *b* = unstandardized coefficient, *SE* = standard error, β = standardized coefficient; all *p*s < .001.

*Factor Loadings of Affective Model for English Non-primary Group*

| Item | *b* | *SE* | β |
| --- | --- | --- | --- |
| *Comfort* |  |  |  |
| Upsetting–Calming | 1.000 |  | 0.672 |
| Distressing–Relaxing | 1.176 | 0.093 | 0.749 |
| Uncomfortable–Comfortable | 1.414 | 0.117 | 0.909 |
| Fearful–Safe | 0.958 | 0.097 | 0.655 |
| *Activity* |  |  |  |
| Inactive–Active | 1.000 |  | 0.740 |
| Sleepy–Arousing | 0.784 | 0.086 | 0.578 |
| Dull–Lively | 1.159 | 0.092 | 0.817 |
| Unstimulating–Stimulating | 0.894 | 0.077 | 0.733 |

*Note*. *b* = unstandardized coefficient, *SE* = standard error, β = standardized coefficient; all *p*s < .001.

*Factor Loadings of Cognitive Model for English Primary Group*

| Item | *b* | *SE* | β |
| --- | --- | --- | --- |
| *Legibility* |  |  |  |
| In this place it would be very easy to find out my way back to any given point. | 1.000 |  | 0.829 |
| In this place it would be very easy to find my way around. | 1.005 | 0.030 | 0.822 |
| In this place it would be very easy to figure out where I am at any given moment. | 0.980 | 0.030 | 0.798 |
| It is very easy to structure and organize this place as a picture. | 0.730 | 0.029 | 0.654 |
| *Enclosure* |  |  |  |
| This place is very stuffy. | 1.000 |  | 0.743 |
| This place is very cramped. | 1.129 | 0.047 | 0.783 |
| In this place I strongly feel being “inside looking out”. | 0.321 | 0.042 | 0.230 |
| This place gives me a strong feeling of being enclosed in a hiding place. | 0.850 | 0.045 | 0.578 |
| *Complexity* |  |  |  |
| There is a lot to look at in this place. | 1.000 |  | 0.826 |
| To a large extent this place promises more to be seen if I could walk deeper in it. | 0.745 | 0.036 | 0.616 |
| This place contains many elements of different kinds. | 0.761 | 0.032 | 0.699 |
| A great deal is going on in this place. | 0.829 | 0.038 | 0.645 |
| *Crime Potential* |  |  |  |
| There are many areas in this place where a potential criminal can hide. | 1.000 |  | 0.791 |
| This place is prone to crimes. | 0.932 | 0.029 | 0.826 |
| There is a large probability that an ill-intentioned person would hide in this place. | 0.996 | 0.030 | 0.836 |
| There is possible danger from other people in this place. | 0.881 | 0.030 | 0.756 |
| *Wildlife* |  |  |  |
| There are many trees, vegetations, and flowers in this place. | 1.000 |  | 0.374 |
| In this place, there is some wildlife that can harm people, such as snakes, bees, and toxic plants. | 1.726 | 0.152 | 0.864 |
| There are potentially harmful animals and plants in this place. | 1.363 | 0.113 | 0.715 |
| *Lighting* |  |  |  |
| This place has uniform lighting. | 1.000 |  | 0.647 |
| The light in this place is very good. | 1.267 | 0.049 | 0.875 |
| This setting has very bright, clear lighting. | 1.286 | 0.051 | 0.860 |

*Note*. *b* = unstandardized coefficient, *SE* = standard error, β = standardized coefficient; all *p*s < .001.

*Factor Loadings of Cognitive Model for English Non-primary Group*

| Item | *b* | *SE* | β |
| --- | --- | --- | --- |
| *Legibility* |  |  |  |
| In this place it would be very easy to find out my way back to any given point. | 1.000 |  | 0.574 |
| In this place it would be very easy to find my way around. | 1.357 | 0.155 | 0.725 |
| In this place it would be very easy to figure out where I am at any given moment. | 1.157 | 0.142 | 0.645 |
| It is very easy to structure and organize this place as a picture. | 1.230 | 0.147 | 0.671 |
| *Enclosure* |  |  |  |
| This place is very stuffy. | 1.000 |  | 0.575 |
| This place is very cramped. | 1.253 | 0.163 | 0.680 |
| In this place I strongly feel being “inside looking out”. | 0.539 | 0.114 | 0.346 |
| This place gives me a strong feeling of being enclosed in a hiding place. | 1.166 | 0.153 | 0.666 |
| *Complexity* |  |  |  |
| There is a lot to look at in this place. | 1.000 |  | 0.656 |
| To a large extent this place promises more to be seen if I could walk deeper in it. | 0.882 | 0.115 | 0.612 |
| This place contains many elements of different kinds. | 0.732 | 0.110 | 0.505 |
| A great deal is going on in this place. | 0.924 | 0.123 | 0.593 |
| *Crime Potential* |  |  |  |
| There are many areas in this place where a potential criminal can hide. | 1.000 |  | 0.654 |
| This place is prone to crimes. | 1.077 | 0.109 | 0.738 |
| There is a large probability that an ill-intentioned person would hide in this place. | 0.973 | 0.104 | 0.684 |
| There is possible danger from other people in this place. | 1.012 | 0.109 | 0.676 |
| *Wildlife* |  |  |  |
| There are many trees, vegetations, and flowers in this place. | 1.000 |  | 0.299 |
| In this place, there is some wildlife that can harm people, such as snakes, bees, and toxic plants. | 2.933 | 0.613 | 0.869 |
| There are potentially harmful animals and plants in this place. | 2.617 | 0.549 | 0.808 |
| *Lighting* |  |  |  |
| This place has uniform lighting. | 1.000 |  | 0.579 |
| The light in this place is very good. | 1.165 | 0.131 | 0.743 |
| This setting has very bright, clear lighting. | 1.201 | 0.135 | 0.747 |

*Note*. *b* = unstandardized coefficient, *SE* = standard error, β = standardized coefficient; all *p*s < .001.
