# Supplementary material for: Scale Development for Environmental Perception of Public Space
Source: Front Psychol. 2020 Nov 23;11:596790. doi: 10.3389/fpsyg.2020.596790 (PMC7719834; doi:10.3389/fpsyg.2020.596790)
Supplement: Supplementary file 3 [file Table_3.DOCX]

Appendix C

**Multiple Comparisons** **with Tukey’s HSD Post-hoc Tests**

**Transport Facility (1 of 2)**

| Dependent Variable | Comparison Space Type | Difference | *SE* | *p* |
| --- | --- | --- | --- | --- |
| Comfort | Street | -0.37 | 0.20 | 0.80000 |
|  | Square | -1.41 | 0.21 | 0.00000 |
|  | Recreational Space | -1.25 | 0.21 | 0.00000 |
|  | Found Neighborhood Space | 0.59 | 0.21 | 0.17400 |
|  | Park | -1.78 | 0.21 | 0.00000 |
|  | Memorial | -0.32 | 0.21 | 0.91700 |
|  | Market | -0.45 | 0.20 | 0.54100 |
|  | Playground | -1.25 | 0.20 | 0.00000 |
|  | Community Open Space | -1.69 | 0.21 | 0.00000 |
|  | Indoor Marketplace | -1.04 | 0.20 | 0.00000 |
|  | Waterfront | -1.45 | 0.20 | 0.00000 |
| Activity | Street | -0.70 | 0.20 | 0.02300 |
|  | Square | -0.60 | 0.20 | 0.13000 |
|  | Recreational Space | -1.01 | 0.21 | 0.00000 |
|  | Found Neighborhood Space | 1.90 | 0.21 | 0.00000 |
|  | Park | -0.28 | 0.21 | 0.97500 |
|  | Memorial | 1.16 | 0.20 | 0.00000 |
|  | Market | -1.29 | 0.20 | 0.00000 |
|  | Playground | -0.92 | 0.20 | 0.00000 |
|  | Community Open Space | 0.25 | 0.21 | 0.98900 |
|  | Indoor Marketplace | -0.87 | 0.20 | 0.00100 |
|  | Waterfront | -0.73 | 0.20 | 0.01700 |
| Legibility | Street | -0.13 | 0.18 | 1.00000 |
|  | Square | -0.80 | 0.19 | 0.00100 |
|  | Recreational Space | -0.74 | 0.19 | 0.00500 |
|  | Found Neighborhood Space | 0.71 | 0.19 | 0.01100 |
|  | Park | -0.43 | 0.19 | 0.51500 |
|  | Memorial | 0.70 | 0.19 | 0.01000 |
|  | Market | 0.57 | 0.19 | 0.09000 |
|  | Playground | -0.62 | 0.18 | 0.03100 |
|  | Community Open Space | -0.63 | 0.19 | 0.04500 |
|  | Indoor Marketplace | -0.25 | 0.18 | 0.97100 |
|  | Waterfront | -0.70 | 0.18 | 0.00700 |
| Enclosure | Street | -0.15 | 0.21 | 1.00000 |
|  | Square | 0.57 | 0.22 | 0.26900 |
|  | Recreational Space | 0.34 | 0.22 | 0.92600 |
|  | Found Neighborhood Space | -0.47 | 0.22 | 0.62000 |
|  | Park | 0.20 | 0.22 | 0.99900 |
|  | Memorial | -0.20 | 0.22 | 0.99900 |
|  | Market | -0.93 | 0.22 | 0.00100 |
|  | Playground | 0.31 | 0.21 | 0.95400 |
|  | Community Open Space | 0.65 | 0.22 | 0.13500 |
|  | Indoor Marketplace | 0.17 | 0.21 | 1.00000 |
|  | Waterfront | 0.60 | 0.21 | 0.18500 |

**Transport Facility (2 of 2)**

| Dependent Variable | Comparison Space Type | Difference | *SE* | *p* |
| --- | --- | --- | --- | --- |
| Complexity | Street | -0.68 | 0.20 | 0.02800 |
|  | Square | -0.04 | 0.20 | 1.00000 |
|  | Recreational Space | 0.52 | 0.20 | 0.28600 |
|  | Found Neighborhood Space | 1.59 | 0.20 | 0.00000 |
|  | Park | -0.10 | 0.20 | 1.00000 |
|  | Memorial | 0.31 | 0.20 | 0.93100 |
|  | Market | -0.96 | 0.20 | 0.00000 |
|  | Playground | -0.04 | 0.19 | 1.00000 |
|  | Community Open Space | 0.26 | 0.21 | 0.98100 |
|  | Indoor Marketplace | -0.87 | 0.20 | 0.00100 |
|  | Waterfront | -0.47 | 0.20 | 0.40800 |
| Crime Potential | Street | 0.56 | 0.22 | 0.32100 |
|  | Square | 1.28 | 0.23 | 0.00000 |
|  | Recreational Space | 0.95 | 0.23 | 0.00200 |
|  | Found Neighborhood Space | -0.88 | 0.23 | 0.00900 |
|  | Park | 1.03 | 0.23 | 0.00100 |
|  | Memorial | 0.31 | 0.23 | 0.96600 |
|  | Market | 0.11 | 0.23 | 1.00000 |
|  | Playground | 1.32 | 0.22 | 0.00000 |
|  | Community Open Space | 1.60 | 0.23 | 0.00000 |
|  | Indoor Marketplace | 0.86 | 0.22 | 0.00700 |
|  | Waterfront | 0.92 | 0.22 | 0.00300 |
| Wildlife | Street | -0.72 | 0.21 | 0.03500 |
|  | Square | -1.48 | 0.22 | 0.00000 |
|  | Recreational Space | -1.65 | 0.22 | 0.00000 |
|  | Found Neighborhood Space | -1.20 | 0.22 | 0.00000 |
|  | Park | -2.14 | 0.22 | 0.00000 |
|  | Memorial | -2.16 | 0.22 | 0.00000 |
|  | Market | -0.93 | 0.22 | 0.00100 |
|  | Playground | -1.31 | 0.21 | 0.00000 |
|  | Community Open Space | -0.82 | 0.22 | 0.01300 |
|  | Indoor Marketplace | -0.03 | 0.21 | 1.00000 |
|  | Waterfront | -0.57 | 0.21 | 0.23500 |
| Lighting | Street | 0.29 | 0.20 | 0.95700 |
|  | Square | -0.30 | 0.21 | 0.95800 |
|  | Recreational Space | -0.15 | 0.21 | 1.00000 |
|  | Found Neighborhood Space | 1.79 | 0.21 | 0.00000 |
|  | Park | 0.08 | 0.21 | 1.00000 |
|  | Memorial | 1.40 | 0.21 | 0.00000 |
|  | Market | 1.08 | 0.21 | 0.00000 |
|  | Playground | -0.03 | 0.20 | 1.00000 |
|  | Community Open Space | -0.38 | 0.21 | 0.82200 |
|  | Indoor Marketplace | -0.43 | 0.20 | 0.59800 |
|  | Waterfront | -0.15 | 0.20 | 1.00000 |

**Street (1 of 2)**

| Dependent Variable | Comparison Space Type | Difference | *SE* | *p* |
| --- | --- | --- | --- | --- |
| Comfort | Transport Facility | 0.37 | 0.20 | 0.80000 |
|  | Square | -1.05 | 0.20 | 0.00000 |
|  | Recreational Space | -0.88 | 0.20 | 0.00100 |
|  | Found Neighborhood Space | 0.96 | 0.20 | 0.00000 |
|  | Park | -1.41 | 0.20 | 0.00000 |
|  | Memorial | 0.04 | 0.20 | 1.00000 |
|  | Market | -0.09 | 0.20 | 1.00000 |
|  | Playground | -0.88 | 0.19 | 0.00000 |
|  | Community Open Space | -1.33 | 0.20 | 0.00000 |
|  | Indoor Marketplace | -0.67 | 0.19 | 0.02800 |
|  | Waterfront | -1.08 | 0.19 | 0.00000 |
| Activity | Transport Facility | 0.70 | 0.20 | 0.02300 |
|  | Square | 0.10 | 0.20 | 1.00000 |
|  | Recreational Space | -0.31 | 0.20 | 0.92600 |
|  | Found Neighborhood Space | 2.60 | 0.20 | 0.00000 |
|  | Park | 0.43 | 0.20 | 0.60400 |
|  | Memorial | 1.87 | 0.20 | 0.00000 |
|  | Market | -0.58 | 0.20 | 0.11900 |
|  | Playground | -0.21 | 0.19 | 0.99400 |
|  | Community Open Space | 0.96 | 0.20 | 0.00000 |
|  | Indoor Marketplace | -0.17 | 0.19 | 0.99900 |
|  | Waterfront | -0.02 | 0.19 | 1.00000 |
| Legibility | Transport Facility | 0.13 | 0.18 | 1.00000 |
|  | Square | -0.68 | 0.18 | 0.00900 |
|  | Recreational Space | -0.61 | 0.18 | 0.03400 |
|  | Found Neighborhood Space | 0.84 | 0.18 | 0.00000 |
|  | Park | -0.30 | 0.18 | 0.89400 |
|  | Memorial | 0.82 | 0.18 | 0.00000 |
|  | Market | 0.70 | 0.18 | 0.00600 |
|  | Playground | -0.49 | 0.17 | 0.16300 |
|  | Community Open Space | -0.51 | 0.18 | 0.20600 |
|  | Indoor Marketplace | -0.12 | 0.18 | 1.00000 |
|  | Waterfront | -0.58 | 0.18 | 0.05100 |
| Enclosure | Transport Facility | 0.15 | 0.21 | 1.00000 |
|  | Square | 0.72 | 0.21 | 0.03000 |
|  | Recreational Space | 0.49 | 0.21 | 0.46000 |
|  | Found Neighborhood Space | -0.32 | 0.21 | 0.94600 |
|  | Park | 0.35 | 0.21 | 0.88600 |
|  | Memorial | -0.05 | 0.21 | 1.00000 |
|  | Market | -0.78 | 0.21 | 0.01000 |
|  | Playground | 0.46 | 0.20 | 0.51700 |
|  | Community Open Space | 0.80 | 0.22 | 0.01100 |
|  | Indoor Marketplace | 0.32 | 0.20 | 0.92400 |
|  | Waterfront | 0.75 | 0.21 | 0.01600 |

**Street (2 of 2)**

| Dependent Variable | Comparison Space Type | Difference | *SE* | *p* |
| --- | --- | --- | --- | --- |
| Complexity | Transport Facility | 0.68 | 0.20 | 0.02800 |
|  | Square | 0.64 | 0.19 | 0.04700 |
|  | Recreational Space | 1.20 | 0.19 | 0.00000 |
|  | Found Neighborhood Space | 2.26 | 0.20 | 0.00000 |
|  | Park | 0.58 | 0.20 | 0.12700 |
|  | Memorial | 0.98 | 0.19 | 0.00000 |
|  | Market | -0.28 | 0.19 | 0.95400 |
|  | Playground | 0.64 | 0.19 | 0.03100 |
|  | Community Open Space | 0.94 | 0.20 | 0.00000 |
|  | Indoor Marketplace | -0.19 | 0.19 | 0.99700 |
|  | Waterfront | 0.20 | 0.19 | 0.99600 |
| Crime Potential | Transport Facility | -0.56 | 0.22 | 0.32100 |
|  | Square | 0.72 | 0.22 | 0.04800 |
|  | Recreational Space | 0.39 | 0.22 | 0.83500 |
|  | Found Neighborhood Space | -1.44 | 0.22 | 0.00000 |
|  | Park | 0.47 | 0.22 | 0.61800 |
|  | Memorial | -0.25 | 0.22 | 0.99300 |
|  | Market | -0.45 | 0.22 | 0.64900 |
|  | Playground | 0.76 | 0.21 | 0.02000 |
|  | Community Open Space | 1.04 | 0.22 | 0.00000 |
|  | Indoor Marketplace | 0.30 | 0.21 | 0.96300 |
|  | Waterfront | 0.36 | 0.22 | 0.88100 |
| Wildlife | Transport Facility | 0.72 | 0.21 | 0.03500 |
|  | Square | -0.76 | 0.21 | 0.01400 |
|  | Recreational Space | -0.93 | 0.21 | 0.00100 |
|  | Found Neighborhood Space | -0.49 | 0.21 | 0.48500 |
|  | Park | -1.43 | 0.21 | 0.00000 |
|  | Memorial | -1.45 | 0.21 | 0.00000 |
|  | Market | -0.21 | 0.21 | 0.99700 |
|  | Playground | -0.59 | 0.20 | 0.13100 |
|  | Community Open Space | -0.10 | 0.21 | 1.00000 |
|  | Indoor Marketplace | 0.69 | 0.20 | 0.03800 |
|  | Waterfront | 0.14 | 0.21 | 1.00000 |
| Lighting | Transport Facility | -0.29 | 0.20 | 0.95700 |
|  | Square | -0.59 | 0.20 | 0.13200 |
|  | Recreational Space | -0.44 | 0.20 | 0.54900 |
|  | Found Neighborhood Space | 1.50 | 0.20 | 0.00000 |
|  | Park | -0.21 | 0.20 | 0.99700 |
|  | Memorial | 1.11 | 0.20 | 0.00000 |
|  | Market | 0.79 | 0.20 | 0.00400 |
|  | Playground | -0.31 | 0.19 | 0.90000 |
|  | Community Open Space | -0.67 | 0.21 | 0.05200 |
|  | Indoor Marketplace | -0.72 | 0.20 | 0.01200 |
|  | Waterfront | -0.44 | 0.20 | 0.53800 |

**Square (1 of 2)**

| Dependent Variable | Comparison Space Type | Difference | *SE* | *p* |
| --- | --- | --- | --- | --- |
| Comfort | Transport Facility | 1.41 | 0.21 | 0.00000 |
|  | Street | 1.05 | 0.20 | 0.00000 |
|  | Recreational Space | 0.17 | 0.20 | 1.00000 |
|  | Found Neighborhood Space | 2.00 | 0.21 | 0.00000 |
|  | Park | -0.37 | 0.21 | 0.83100 |
|  | Memorial | 1.09 | 0.20 | 0.00000 |
|  | Market | 0.96 | 0.20 | 0.00000 |
|  | Playground | 0.17 | 0.20 | 1.00000 |
|  | Community Open Space | -0.28 | 0.21 | 0.97300 |
|  | Indoor Marketplace | 0.38 | 0.20 | 0.75800 |
|  | Waterfront | -0.04 | 0.20 | 1.00000 |
| Activity | Transport Facility | 0.60 | 0.20 | 0.13000 |
|  | Street | -0.10 | 0.20 | 1.00000 |
|  | Recreational Space | -0.41 | 0.20 | 0.68000 |
|  | Found Neighborhood Space | 2.50 | 0.21 | 0.00000 |
|  | Park | 0.32 | 0.21 | 0.91700 |
|  | Memorial | 1.76 | 0.20 | 0.00000 |
|  | Market | -0.69 | 0.20 | 0.03300 |
|  | Playground | -0.32 | 0.20 | 0.90600 |
|  | Community Open Space | 0.85 | 0.21 | 0.00300 |
|  | Indoor Marketplace | -0.27 | 0.20 | 0.96700 |
|  | Waterfront | -0.13 | 0.20 | 1.00000 |
| Legibility | Transport Facility | 0.80 | 0.19 | 0.00100 |
|  | Street | 0.68 | 0.18 | 0.00900 |
|  | Recreational Space | 0.07 | 0.18 | 1.00000 |
|  | Found Neighborhood Space | 1.51 | 0.19 | 0.00000 |
|  | Park | 0.38 | 0.19 | 0.67500 |
|  | Memorial | 1.50 | 0.18 | 0.00000 |
|  | Market | 1.37 | 0.18 | 0.00000 |
|  | Playground | 0.18 | 0.18 | 0.99700 |
|  | Community Open Space | 0.17 | 0.19 | 0.99900 |
|  | Indoor Marketplace | 0.56 | 0.18 | 0.08400 |
|  | Waterfront | 0.10 | 0.18 | 1.00000 |
| Enclosure | Transport Facility | -0.57 | 0.22 | 0.26900 |
|  | Street | -0.72 | 0.21 | 0.03000 |
|  | Recreational Space | -0.23 | 0.22 | 0.99600 |
|  | Found Neighborhood Space | -1.04 | 0.22 | 0.00000 |
|  | Park | -0.37 | 0.22 | 0.87800 |
|  | Memorial | -0.77 | 0.21 | 0.01800 |
|  | Market | -1.50 | 0.21 | 0.00000 |
|  | Playground | -0.26 | 0.21 | 0.98300 |
|  | Community Open Space | 0.08 | 0.22 | 1.00000 |
|  | Indoor Marketplace | -0.40 | 0.21 | 0.75300 |
|  | Waterfront | 0.03 | 0.21 | 1.00000 |

**Square (2 of 2)**

| Dependent Variable | Comparison Space Type | Difference | *SE* | *p* |
| --- | --- | --- | --- | --- |
| Complexity | Transport Facility | 0.04 | 0.20 | 1.00000 |
|  | Street | -0.64 | 0.19 | 0.04700 |
|  | Recreational Space | 0.56 | 0.20 | 0.16500 |
|  | Found Neighborhood Space | 1.63 | 0.20 | 0.00000 |
|  | Park | -0.06 | 0.20 | 1.00000 |
|  | Memorial | 0.35 | 0.20 | 0.83500 |
|  | Market | -0.91 | 0.20 | 0.00000 |
|  | Playground | 0.01 | 0.19 | 1.00000 |
|  | Community Open Space | 0.31 | 0.20 | 0.93800 |
|  | Indoor Marketplace | -0.83 | 0.19 | 0.00100 |
|  | Waterfront | -0.43 | 0.20 | 0.54000 |
| Crime Potential | Transport Facility | -1.28 | 0.23 | 0.00000 |
|  | Street | -0.72 | 0.22 | 0.04800 |
|  | Recreational Space | -0.33 | 0.22 | 0.94700 |
|  | Found Neighborhood Space | -2.16 | 0.23 | 0.00000 |
|  | Park | -0.25 | 0.23 | 0.99500 |
|  | Memorial | -0.97 | 0.22 | 0.00100 |
|  | Market | -1.17 | 0.22 | 0.00000 |
|  | Playground | 0.04 | 0.22 | 1.00000 |
|  | Community Open Space | 0.32 | 0.23 | 0.96700 |
|  | Indoor Marketplace | -0.42 | 0.22 | 0.75300 |
|  | Waterfront | -0.36 | 0.22 | 0.90000 |
| Wildlife | Transport Facility | 1.48 | 0.22 | 0.00000 |
|  | Street | 0.76 | 0.21 | 0.01400 |
|  | Recreational Space | -0.17 | 0.21 | 1.00000 |
|  | Found Neighborhood Space | 0.28 | 0.22 | 0.98300 |
|  | Park | -0.66 | 0.22 | 0.09500 |
|  | Memorial | -0.68 | 0.21 | 0.06200 |
|  | Market | 0.55 | 0.21 | 0.28200 |
|  | Playground | 0.17 | 0.21 | 1.00000 |
|  | Community Open Space | 0.66 | 0.22 | 0.10500 |
|  | Indoor Marketplace | 1.45 | 0.21 | 0.00000 |
|  | Waterfront | 0.91 | 0.21 | 0.00100 |
| Lighting | Transport Facility | 0.30 | 0.21 | 0.95800 |
|  | Street | 0.59 | 0.20 | 0.13200 |
|  | Recreational Space | 0.14 | 0.21 | 1.00000 |
|  | Found Neighborhood Space | 2.08 | 0.21 | 0.00000 |
|  | Park | 0.38 | 0.21 | 0.80600 |
|  | Memorial | 1.70 | 0.20 | 0.00000 |
|  | Market | 1.38 | 0.20 | 0.00000 |
|  | Playground | 0.27 | 0.20 | 0.97000 |
|  | Community Open Space | -0.09 | 0.21 | 1.00000 |
|  | Indoor Marketplace | -0.14 | 0.20 | 1.00000 |
|  | Waterfront | 0.15 | 0.20 | 1.00000 |

**Recreational Space (1 of 2)**

| Dependent Variable | Comparison Space Type | Difference | *SE* | *p* |
| --- | --- | --- | --- | --- |
| Comfort | Transport Facility | 1.25 | 0.21 | 0.00000 |
|  | Street | 0.88 | 0.20 | 0.00100 |
|  | Square | -0.17 | 0.20 | 1.00000 |
|  | Found Neighborhood Space | 1.84 | 0.21 | 0.00000 |
|  | Park | -0.53 | 0.21 | 0.29700 |
|  | Memorial | 0.92 | 0.20 | 0.00000 |
|  | Market | 0.80 | 0.20 | 0.00500 |
|  | Playground | 0.00 | 0.20 | 1.00000 |
|  | Community Open Space | -0.45 | 0.21 | 0.59900 |
|  | Indoor Marketplace | 0.21 | 0.20 | 0.99600 |
|  | Waterfront | -0.20 | 0.20 | 0.99700 |
| Activity | Transport Facility | 1.01 | 0.21 | 0.00000 |
|  | Street | 0.31 | 0.20 | 0.92600 |
|  | Square | 0.41 | 0.20 | 0.68000 |
|  | Found Neighborhood Space | 2.91 | 0.21 | 0.00000 |
|  | Park | 0.73 | 0.21 | 0.02100 |
|  | Memorial | 2.17 | 0.20 | 0.00000 |
|  | Market | -0.28 | 0.20 | 0.96900 |
|  | Playground | 0.09 | 0.20 | 1.00000 |
|  | Community Open Space | 1.26 | 0.21 | 0.00000 |
|  | Indoor Marketplace | 0.14 | 0.20 | 1.00000 |
|  | Waterfront | 0.28 | 0.20 | 0.96200 |
| Legibility | Transport Facility | 0.74 | 0.19 | 0.00500 |
|  | Street | 0.61 | 0.18 | 0.03400 |
|  | Square | -0.07 | 0.18 | 1.00000 |
|  | Found Neighborhood Space | 1.45 | 0.19 | 0.00000 |
|  | Park | 0.31 | 0.19 | 0.88200 |
|  | Memorial | 1.43 | 0.18 | 0.00000 |
|  | Market | 1.31 | 0.18 | 0.00000 |
|  | Playground | 0.12 | 0.18 | 1.00000 |
|  | Community Open Space | 0.11 | 0.19 | 1.00000 |
|  | Indoor Marketplace | 0.49 | 0.18 | 0.21700 |
|  | Waterfront | 0.03 | 0.18 | 1.00000 |
| Enclosure | Transport Facility | -0.34 | 0.22 | 0.92600 |
|  | Street | -0.49 | 0.21 | 0.46000 |
|  | Square | 0.23 | 0.22 | 0.99600 |
|  | Found Neighborhood Space | -0.81 | 0.22 | 0.01400 |
|  | Park | -0.13 | 0.22 | 1.00000 |
|  | Memorial | -0.54 | 0.22 | 0.33600 |
|  | Market | -1.27 | 0.21 | 0.00000 |
|  | Playground | -0.03 | 0.21 | 1.00000 |
|  | Community Open Space | 0.31 | 0.22 | 0.95900 |
|  | Indoor Marketplace | -0.17 | 0.21 | 1.00000 |
|  | Waterfront | 0.26 | 0.21 | 0.98600 |

**Recreational Space (2 of 2)**

| Dependent Variable | Comparison Space Type | Difference | *SE* | *p* |
| --- | --- | --- | --- | --- |
| Complexity | Transport Facility | -0.52 | 0.20 | 0.28600 |
|  | Street | -1.20 | 0.19 | 0.00000 |
|  | Square | -0.56 | 0.20 | 0.16500 |
|  | Found Neighborhood Space | 1.07 | 0.20 | 0.00000 |
|  | Park | -0.62 | 0.20 | 0.09200 |
|  | Memorial | -0.21 | 0.20 | 0.99500 |
|  | Market | -1.48 | 0.20 | 0.00000 |
|  | Playground | -0.56 | 0.19 | 0.14900 |
|  | Community Open Space | -0.26 | 0.20 | 0.98300 |
|  | Indoor Marketplace | -1.39 | 0.19 | 0.00000 |
|  | Waterfront | -1.00 | 0.20 | 0.00000 |
| Crime Potential | Transport Facility | -0.95 | 0.23 | 0.00200 |
|  | Street | -0.39 | 0.22 | 0.83500 |
|  | Square | 0.33 | 0.22 | 0.94700 |
|  | Found Neighborhood Space | -1.83 | 0.23 | 0.00000 |
|  | Park | 0.08 | 0.23 | 1.00000 |
|  | Memorial | -0.63 | 0.22 | 0.17200 |
|  | Market | -0.84 | 0.22 | 0.01100 |
|  | Playground | 0.37 | 0.22 | 0.87600 |
|  | Community Open Space | 0.65 | 0.23 | 0.17900 |
|  | Indoor Marketplace | -0.09 | 0.22 | 1.00000 |
|  | Waterfront | -0.03 | 0.22 | 1.00000 |
| Wildlife | Transport Facility | 1.65 | 0.22 | 0.00000 |
|  | Street | 0.93 | 0.21 | 0.00100 |
|  | Square | 0.17 | 0.21 | 1.00000 |
|  | Found Neighborhood Space | 0.44 | 0.22 | 0.67600 |
|  | Park | -0.50 | 0.22 | 0.49400 |
|  | Memorial | -0.52 | 0.21 | 0.39800 |
|  | Market | 0.72 | 0.21 | 0.03700 |
|  | Playground | 0.34 | 0.21 | 0.90000 |
|  | Community Open Space | 0.83 | 0.22 | 0.01000 |
|  | Indoor Marketplace | 1.62 | 0.21 | 0.00000 |
|  | Waterfront | 1.07 | 0.21 | 0.00000 |
| Lighting | Transport Facility | 0.15 | 0.21 | 1.00000 |
|  | Street | 0.44 | 0.20 | 0.54900 |
|  | Square | -0.14 | 0.21 | 1.00000 |
|  | Found Neighborhood Space | 1.94 | 0.21 | 0.00000 |
|  | Park | 0.24 | 0.21 | 0.99400 |
|  | Memorial | 1.55 | 0.21 | 0.00000 |
|  | Market | 1.23 | 0.20 | 0.00000 |
|  | Playground | 0.13 | 0.20 | 1.00000 |
|  | Community Open Space | -0.23 | 0.21 | 0.99500 |
|  | Indoor Marketplace | -0.28 | 0.20 | 0.96400 |
|  | Waterfront | 0.00 | 0.20 | 1.00000 |

**Found Neighborhood Space (1 of 2)**

| Dependent Variable | Comparison Space Type | Difference | *SE* | *p* |
| --- | --- | --- | --- | --- |
| Comfort | Transport Facility | -0.59 | 0.21 | 0.17400 |
|  | Street | -0.96 | 0.20 | 0.00000 |
|  | Square | -2.00 | 0.21 | 0.00000 |
|  | Recreational Space | -1.84 | 0.21 | 0.00000 |
|  | Park | -2.37 | 0.21 | 0.00000 |
|  | Memorial | -0.91 | 0.21 | 0.00100 |
|  | Market | -1.04 | 0.21 | 0.00000 |
|  | Playground | -1.84 | 0.20 | 0.00000 |
|  | Community Open Space | -2.28 | 0.21 | 0.00000 |
|  | Indoor Marketplace | -1.63 | 0.20 | 0.00000 |
|  | Waterfront | -2.04 | 0.20 | 0.00000 |
| Activity | Transport Facility | -1.90 | 0.21 | 0.00000 |
|  | Street | -2.60 | 0.20 | 0.00000 |
|  | Square | -2.50 | 0.21 | 0.00000 |
|  | Recreational Space | -2.91 | 0.21 | 0.00000 |
|  | Park | -2.17 | 0.21 | 0.00000 |
|  | Memorial | -0.74 | 0.21 | 0.02000 |
|  | Market | -3.18 | 0.21 | 0.00000 |
|  | Playground | -2.81 | 0.20 | 0.00000 |
|  | Community Open Space | -1.65 | 0.21 | 0.00000 |
|  | Indoor Marketplace | -2.77 | 0.20 | 0.00000 |
|  | Waterfront | -2.63 | 0.20 | 0.00000 |
| Legibility | Transport Facility | -0.71 | 0.19 | 0.01100 |
|  | Street | -0.84 | 0.18 | 0.00000 |
|  | Square | -1.51 | 0.19 | 0.00000 |
|  | Recreational Space | -1.45 | 0.19 | 0.00000 |
|  | Park | -1.13 | 0.19 | 0.00000 |
|  | Memorial | -0.01 | 0.19 | 1.00000 |
|  | Market | -0.14 | 0.19 | 1.00000 |
|  | Playground | -1.33 | 0.18 | 0.00000 |
|  | Community Open Space | -1.34 | 0.19 | 0.00000 |
|  | Indoor Marketplace | -0.96 | 0.18 | 0.00000 |
|  | Waterfront | -1.41 | 0.19 | 0.00000 |
| Enclosure | Transport Facility | 0.47 | 0.22 | 0.62000 |
|  | Street | 0.32 | 0.21 | 0.94600 |
|  | Square | 1.04 | 0.22 | 0.00000 |
|  | Recreational Space | 0.81 | 0.22 | 0.01400 |
|  | Park | 0.67 | 0.22 | 0.10900 |
|  | Memorial | 0.27 | 0.22 | 0.98800 |
|  | Market | -0.46 | 0.22 | 0.60700 |
|  | Playground | 0.77 | 0.21 | 0.01600 |
|  | Community Open Space | 1.12 | 0.23 | 0.00000 |
|  | Indoor Marketplace | 0.64 | 0.22 | 0.12400 |
|  | Waterfront | 1.07 | 0.22 | 0.00000 |

**Found Neighborhood Space (2 of 2)**

| Dependent Variable | Comparison Space Type | Difference | *SE* | *p* |
| --- | --- | --- | --- | --- |
| Complexity | Transport Facility | -1.59 | 0.20 | 0.00000 |
|  | Street | -2.26 | 0.20 | 0.00000 |
|  | Square | -1.63 | 0.20 | 0.00000 |
|  | Recreational Space | -1.07 | 0.20 | 0.00000 |
|  | Park | -1.69 | 0.21 | 0.00000 |
|  | Memorial | -1.28 | 0.20 | 0.00000 |
|  | Market | -2.54 | 0.20 | 0.00000 |
|  | Playground | -1.62 | 0.20 | 0.00000 |
|  | Community Open Space | -1.32 | 0.21 | 0.00000 |
|  | Indoor Marketplace | -2.45 | 0.20 | 0.00000 |
|  | Waterfront | -2.06 | 0.20 | 0.00000 |
| Crime Potential | Transport Facility | 0.88 | 0.23 | 0.00900 |
|  | Street | 1.44 | 0.22 | 0.00000 |
|  | Square | 2.16 | 0.23 | 0.00000 |
|  | Recreational Space | 1.83 | 0.23 | 0.00000 |
|  | Park | 1.91 | 0.23 | 0.00000 |
|  | Memorial | 1.19 | 0.23 | 0.00000 |
|  | Market | 0.99 | 0.23 | 0.00100 |
|  | Playground | 2.19 | 0.22 | 0.00000 |
|  | Community Open Space | 2.48 | 0.24 | 0.00000 |
|  | Indoor Marketplace | 1.74 | 0.22 | 0.00000 |
|  | Waterfront | 1.80 | 0.23 | 0.00000 |
| Wildlife | Transport Facility | 1.20 | 0.22 | 0.00000 |
|  | Street | 0.49 | 0.21 | 0.48500 |
|  | Square | -0.28 | 0.22 | 0.98300 |
|  | Recreational Space | -0.44 | 0.22 | 0.67600 |
|  | Park | -0.94 | 0.22 | 0.00200 |
|  | Memorial | -0.96 | 0.22 | 0.00100 |
|  | Market | 0.28 | 0.22 | 0.98300 |
|  | Playground | -0.11 | 0.21 | 1.00000 |
|  | Community Open Space | 0.39 | 0.22 | 0.85700 |
|  | Indoor Marketplace | 1.17 | 0.21 | 0.00000 |
|  | Waterfront | 0.63 | 0.22 | 0.13400 |
| Lighting | Transport Facility | -1.79 | 0.21 | 0.00000 |
|  | Street | -1.50 | 0.20 | 0.00000 |
|  | Square | -2.08 | 0.21 | 0.00000 |
|  | Recreational Space | -1.94 | 0.21 | 0.00000 |
|  | Park | -1.70 | 0.21 | 0.00000 |
|  | Memorial | -0.39 | 0.21 | 0.79100 |
|  | Market | -0.71 | 0.21 | 0.03500 |
|  | Playground | -1.81 | 0.20 | 0.00000 |
|  | Community Open Space | -2.17 | 0.21 | 0.00000 |
|  | Indoor Marketplace | -2.22 | 0.21 | 0.00000 |
|  | Waterfront | -1.93 | 0.21 | 0.00000 |

**Park (1 of 2)**

| Dependent Variable | Comparison Space Type | Difference | *SE* | *p* |
| --- | --- | --- | --- | --- |
| Comfort | Transport Facility | 1.78 | 0.21 | 0.00000 |
|  | Street | 1.41 | 0.20 | 0.00000 |
|  | Square | 0.37 | 0.21 | 0.83100 |
|  | Recreational Space | 0.53 | 0.21 | 0.29700 |
|  | Found Neighborhood Space | 2.37 | 0.21 | 0.00000 |
|  | Memorial | 1.46 | 0.21 | 0.00000 |
|  | Market | 1.33 | 0.21 | 0.00000 |
|  | Playground | 0.53 | 0.20 | 0.25500 |
|  | Community Open Space | 0.09 | 0.21 | 1.00000 |
|  | Indoor Marketplace | 0.74 | 0.20 | 0.01300 |
|  | Waterfront | 0.33 | 0.20 | 0.90300 |
| Activity | Transport Facility | 0.28 | 0.21 | 0.97500 |
|  | Street | -0.43 | 0.20 | 0.60400 |
|  | Square | -0.32 | 0.21 | 0.91700 |
|  | Recreational Space | -0.73 | 0.21 | 0.02100 |
|  | Found Neighborhood Space | 2.17 | 0.21 | 0.00000 |
|  | Memorial | 1.44 | 0.21 | 0.00000 |
|  | Market | -1.01 | 0.20 | 0.00000 |
|  | Playground | -0.64 | 0.20 | 0.06400 |
|  | Community Open Space | 0.53 | 0.21 | 0.33700 |
|  | Indoor Marketplace | -0.60 | 0.20 | 0.12300 |
|  | Waterfront | -0.45 | 0.20 | 0.53200 |
| Legibility | Transport Facility | 0.43 | 0.19 | 0.51500 |
|  | Street | 0.30 | 0.18 | 0.89400 |
|  | Square | -0.38 | 0.19 | 0.67500 |
|  | Recreational Space | -0.31 | 0.19 | 0.88200 |
|  | Found Neighborhood Space | 1.13 | 0.19 | 0.00000 |
|  | Memorial | 1.12 | 0.19 | 0.00000 |
|  | Market | 0.99 | 0.19 | 0.00000 |
|  | Playground | -0.20 | 0.18 | 0.99600 |
|  | Community Open Space | -0.21 | 0.19 | 0.99500 |
|  | Indoor Marketplace | 0.18 | 0.18 | 0.99800 |
|  | Waterfront | -0.28 | 0.18 | 0.93700 |
| Enclosure | Transport Facility | -0.20 | 0.22 | 0.99900 |
|  | Street | -0.35 | 0.21 | 0.88600 |
|  | Square | 0.37 | 0.22 | 0.87800 |
|  | Recreational Space | 0.13 | 0.22 | 1.00000 |
|  | Found Neighborhood Space | -0.67 | 0.22 | 0.10900 |
|  | Memorial | -0.41 | 0.22 | 0.78600 |
|  | Market | -1.13 | 0.22 | 0.00000 |
|  | Playground | 0.10 | 0.21 | 1.00000 |
|  | Community Open Space | 0.45 | 0.22 | 0.69000 |
|  | Indoor Marketplace | -0.04 | 0.21 | 1.00000 |
|  | Waterfront | 0.40 | 0.22 | 0.79700 |

**Park (2 of 2)**

| Dependent Variable | Comparison Space Type | Difference | *SE* | *p* |
| --- | --- | --- | --- | --- |
| Complexity | Transport Facility | 0.10 | 0.20 | 1.00000 |
|  | Street | -0.58 | 0.20 | 0.12700 |
|  | Square | 0.06 | 0.20 | 1.00000 |
|  | Recreational Space | 0.62 | 0.20 | 0.09200 |
|  | Found Neighborhood Space | 1.69 | 0.21 | 0.00000 |
|  | Memorial | 0.41 | 0.20 | 0.68100 |
|  | Market | -0.86 | 0.20 | 0.00100 |
|  | Playground | 0.06 | 0.20 | 1.00000 |
|  | Community Open Space | 0.36 | 0.21 | 0.84100 |
|  | Indoor Marketplace | -0.77 | 0.20 | 0.00600 |
|  | Waterfront | -0.38 | 0.20 | 0.76600 |
| Crime Potential | Transport Facility | -1.03 | 0.23 | 0.00100 |
|  | Street | -0.47 | 0.22 | 0.61800 |
|  | Square | 0.25 | 0.23 | 0.99500 |
|  | Recreational Space | -0.08 | 0.23 | 1.00000 |
|  | Found Neighborhood Space | -1.91 | 0.23 | 0.00000 |
|  | Memorial | -0.72 | 0.23 | 0.07500 |
|  | Market | -0.92 | 0.23 | 0.00300 |
|  | Playground | 0.29 | 0.22 | 0.98000 |
|  | Community Open Space | 0.57 | 0.23 | 0.39100 |
|  | Indoor Marketplace | -0.17 | 0.22 | 1.00000 |
|  | Waterfront | -0.11 | 0.23 | 1.00000 |
| Wildlife | Transport Facility | 2.14 | 0.22 | 0.00000 |
|  | Street | 1.43 | 0.21 | 0.00000 |
|  | Square | 0.66 | 0.22 | 0.09500 |
|  | Recreational Space | 0.50 | 0.22 | 0.49400 |
|  | Found Neighborhood Space | 0.94 | 0.22 | 0.00200 |
|  | Memorial | -0.02 | 0.22 | 1.00000 |
|  | Market | 1.22 | 0.22 | 0.00000 |
|  | Playground | 0.83 | 0.21 | 0.00500 |
|  | Community Open Space | 1.33 | 0.22 | 0.00000 |
|  | Indoor Marketplace | 2.11 | 0.21 | 0.00000 |
|  | Waterfront | 1.57 | 0.21 | 0.00000 |
| Lighting | Transport Facility | -0.08 | 0.21 | 1.00000 |
|  | Street | 0.21 | 0.20 | 0.99700 |
|  | Square | -0.38 | 0.21 | 0.80600 |
|  | Recreational Space | -0.24 | 0.21 | 0.99400 |
|  | Found Neighborhood Space | 1.70 | 0.21 | 0.00000 |
|  | Memorial | 1.32 | 0.21 | 0.00000 |
|  | Market | 1.00 | 0.21 | 0.00000 |
|  | Playground | -0.11 | 0.20 | 1.00000 |
|  | Community Open Space | -0.46 | 0.21 | 0.56900 |
|  | Indoor Marketplace | -0.52 | 0.20 | 0.32200 |
|  | Waterfront | -0.23 | 0.21 | 0.99400 |

**Memorial (1 of 2)**

| Dependent Variable | Comparison Space Type | Difference | *SE* | *p* |
| --- | --- | --- | --- | --- |
| Comfort | Transport Facility | 0.32 | 0.21 | 0.91700 |
|  | Street | -0.04 | 0.20 | 1.00000 |
|  | Square | -1.09 | 0.20 | 0.00000 |
|  | Recreational Space | -0.92 | 0.20 | 0.00000 |
|  | Found Neighborhood Space | 0.91 | 0.21 | 0.00100 |
|  | Park | -1.46 | 0.21 | 0.00000 |
|  | Market | -0.13 | 0.20 | 1.00000 |
|  | Playground | -0.93 | 0.20 | 0.00000 |
|  | Community Open Space | -1.37 | 0.21 | 0.00000 |
|  | Indoor Marketplace | -0.71 | 0.20 | 0.01800 |
|  | Waterfront | -1.13 | 0.20 | 0.00000 |
| Activity | Transport Facility | -1.16 | 0.20 | 0.00000 |
|  | Street | -1.87 | 0.20 | 0.00000 |
|  | Square | -1.76 | 0.20 | 0.00000 |
|  | Recreational Space | -2.17 | 0.20 | 0.00000 |
|  | Found Neighborhood Space | 0.74 | 0.21 | 0.02000 |
|  | Park | -1.44 | 0.21 | 0.00000 |
|  | Market | -2.45 | 0.20 | 0.00000 |
|  | Playground | -2.08 | 0.20 | 0.00000 |
|  | Community Open Space | -0.91 | 0.21 | 0.00100 |
|  | Indoor Marketplace | -2.04 | 0.20 | 0.00000 |
|  | Waterfront | -1.89 | 0.20 | 0.00000 |
| Legibility | Transport Facility | -0.70 | 0.19 | 0.01000 |
|  | Street | -0.82 | 0.18 | 0.00000 |
|  | Square | -1.50 | 0.18 | 0.00000 |
|  | Recreational Space | -1.43 | 0.18 | 0.00000 |
|  | Found Neighborhood Space | 0.01 | 0.19 | 1.00000 |
|  | Park | -1.12 | 0.19 | 0.00000 |
|  | Market | -0.13 | 0.18 | 1.00000 |
|  | Playground | -1.32 | 0.18 | 0.00000 |
|  | Community Open Space | -1.33 | 0.19 | 0.00000 |
|  | Indoor Marketplace | -0.94 | 0.18 | 0.00000 |
|  | Waterfront | -1.40 | 0.18 | 0.00000 |
| Enclosure | Transport Facility | 0.20 | 0.22 | 0.99900 |
|  | Street | 0.05 | 0.21 | 1.00000 |
|  | Square | 0.77 | 0.21 | 0.01800 |
|  | Recreational Space | 0.54 | 0.22 | 0.33600 |
|  | Found Neighborhood Space | -0.27 | 0.22 | 0.98800 |
|  | Park | 0.41 | 0.22 | 0.78600 |
|  | Market | -0.73 | 0.21 | 0.03300 |
|  | Playground | 0.51 | 0.21 | 0.38400 |
|  | Community Open Space | 0.85 | 0.22 | 0.00600 |
|  | Indoor Marketplace | 0.37 | 0.21 | 0.83800 |
|  | Waterfront | 0.80 | 0.21 | 0.00900 |

**Memorial (2 of 2)**

| Dependent Variable | Comparison Space Type | Difference | *SE* | *p* |
| --- | --- | --- | --- | --- |
| Complexity | Transport Facility | -0.31 | 0.20 | 0.93100 |
|  | Street | -0.98 | 0.19 | 0.00000 |
|  | Square | -0.35 | 0.20 | 0.83500 |
|  | Recreational Space | 0.21 | 0.20 | 0.99500 |
|  | Found Neighborhood Space | 1.28 | 0.20 | 0.00000 |
|  | Park | -0.41 | 0.20 | 0.68100 |
|  | Market | -1.26 | 0.20 | 0.00000 |
|  | Playground | -0.34 | 0.19 | 0.82800 |
|  | Community Open Space | -0.04 | 0.20 | 1.00000 |
|  | Indoor Marketplace | -1.18 | 0.19 | 0.00000 |
|  | Waterfront | -0.78 | 0.20 | 0.00400 |
| Crime Potential | Transport Facility | -0.31 | 0.23 | 0.96600 |
|  | Street | 0.25 | 0.22 | 0.99300 |
|  | Square | 0.97 | 0.22 | 0.00100 |
|  | Recreational Space | 0.63 | 0.22 | 0.17200 |
|  | Found Neighborhood Space | -1.19 | 0.23 | 0.00000 |
|  | Park | 0.72 | 0.23 | 0.07500 |
|  | Market | -0.20 | 0.22 | 0.99900 |
|  | Playground | 1.00 | 0.22 | 0.00000 |
|  | Community Open Space | 1.28 | 0.23 | 0.00000 |
|  | Indoor Marketplace | 0.55 | 0.22 | 0.34500 |
|  | Waterfront | 0.61 | 0.22 | 0.20600 |
| Wildlife | Transport Facility | 2.16 | 0.22 | 0.00000 |
|  | Street | 1.45 | 0.21 | 0.00000 |
|  | Square | 0.68 | 0.21 | 0.06200 |
|  | Recreational Space | 0.52 | 0.21 | 0.39800 |
|  | Found Neighborhood Space | 0.96 | 0.22 | 0.00100 |
|  | Park | 0.02 | 0.22 | 1.00000 |
|  | Market | 1.24 | 0.21 | 0.00000 |
|  | Playground | 0.85 | 0.21 | 0.00200 |
|  | Community Open Space | 1.35 | 0.22 | 0.00000 |
|  | Indoor Marketplace | 2.13 | 0.21 | 0.00000 |
|  | Waterfront | 1.59 | 0.21 | 0.00000 |
| Lighting | Transport Facility | -1.40 | 0.21 | 0.00000 |
|  | Street | -1.11 | 0.20 | 0.00000 |
|  | Square | -1.70 | 0.20 | 0.00000 |
|  | Recreational Space | -1.55 | 0.21 | 0.00000 |
|  | Found Neighborhood Space | 0.39 | 0.21 | 0.79100 |
|  | Park | -1.32 | 0.21 | 0.00000 |
|  | Market | -0.32 | 0.20 | 0.91800 |
|  | Playground | -1.42 | 0.20 | 0.00000 |
|  | Community Open Space | -1.78 | 0.21 | 0.00000 |
|  | Indoor Marketplace | -1.83 | 0.20 | 0.00000 |
|  | Waterfront | -1.55 | 0.20 | 0.00000 |

**Market (1 of 2)**

| Dependent Variable | Comparison Space Type | Difference | *SE* | *p* |
| --- | --- | --- | --- | --- |
| Comfort | Transport Facility | 0.45 | 0.20 | 0.54100 |
|  | Street | 0.09 | 0.20 | 1.00000 |
|  | Square | -0.96 | 0.20 | 0.00000 |
|  | Recreational Space | -0.80 | 0.20 | 0.00500 |
|  | Found Neighborhood Space | 1.04 | 0.21 | 0.00000 |
|  | Park | -1.33 | 0.21 | 0.00000 |
|  | Memorial | 0.13 | 0.20 | 1.00000 |
|  | Playground | -0.80 | 0.20 | 0.00300 |
|  | Community Open Space | -1.24 | 0.21 | 0.00000 |
|  | Indoor Marketplace | -0.58 | 0.20 | 0.12200 |
|  | Waterfront | -1.00 | 0.20 | 0.00000 |
| Activity | Transport Facility | 1.29 | 0.20 | 0.00000 |
|  | Street | 0.58 | 0.20 | 0.11900 |
|  | Square | 0.69 | 0.20 | 0.03300 |
|  | Recreational Space | 0.28 | 0.20 | 0.96900 |
|  | Found Neighborhood Space | 3.18 | 0.21 | 0.00000 |
|  | Park | 1.01 | 0.20 | 0.00000 |
|  | Memorial | 2.45 | 0.20 | 0.00000 |
|  | Playground | 0.37 | 0.20 | 0.76300 |
|  | Community Open Space | 1.54 | 0.21 | 0.00000 |
|  | Indoor Marketplace | 0.41 | 0.20 | 0.62400 |
|  | Waterfront | 0.56 | 0.20 | 0.17700 |
| Legibility | Transport Facility | -0.57 | 0.19 | 0.09000 |
|  | Street | -0.70 | 0.18 | 0.00600 |
|  | Square | -1.37 | 0.18 | 0.00000 |
|  | Recreational Space | -1.31 | 0.18 | 0.00000 |
|  | Found Neighborhood Space | 0.14 | 0.19 | 1.00000 |
|  | Park | -0.99 | 0.19 | 0.00000 |
|  | Memorial | 0.13 | 0.18 | 1.00000 |
|  | Playground | -1.19 | 0.18 | 0.00000 |
|  | Community Open Space | -1.20 | 0.19 | 0.00000 |
|  | Indoor Marketplace | -0.82 | 0.18 | 0.00000 |
|  | Waterfront | -1.27 | 0.18 | 0.00000 |
| Enclosure | Transport Facility | 0.93 | 0.22 | 0.00100 |
|  | Street | 0.78 | 0.21 | 0.01000 |
|  | Square | 1.50 | 0.21 | 0.00000 |
|  | Recreational Space | 1.27 | 0.21 | 0.00000 |
|  | Found Neighborhood Space | 0.46 | 0.22 | 0.60700 |
|  | Park | 1.13 | 0.22 | 0.00000 |
|  | Memorial | 0.73 | 0.21 | 0.03300 |
|  | Playground | 1.24 | 0.21 | 0.00000 |
|  | Community Open Space | 1.58 | 0.22 | 0.00000 |
|  | Indoor Marketplace | 1.10 | 0.21 | 0.00000 |
|  | Waterfront | 1.53 | 0.21 | 0.00000 |

**Market (2 of 2)**

| Dependent Variable | Comparison Space Type | Difference | *SE* | *p* |
| --- | --- | --- | --- | --- |
| Complexity | Transport Facility | 0.96 | 0.20 | 0.00000 |
|  | Street | 0.28 | 0.19 | 0.95400 |
|  | Square | 0.91 | 0.20 | 0.00000 |
|  | Recreational Space | 1.48 | 0.20 | 0.00000 |
|  | Found Neighborhood Space | 2.54 | 0.20 | 0.00000 |
|  | Park | 0.86 | 0.20 | 0.00100 |
|  | Memorial | 1.26 | 0.20 | 0.00000 |
|  | Playground | 0.92 | 0.19 | 0.00000 |
|  | Community Open Space | 1.22 | 0.20 | 0.00000 |
|  | Indoor Marketplace | 0.09 | 0.19 | 1.00000 |
|  | Waterfront | 0.48 | 0.19 | 0.35500 |
| Crime Potential | Transport Facility | -0.11 | 0.23 | 1.00000 |
|  | Street | 0.45 | 0.22 | 0.64900 |
|  | Square | 1.17 | 0.22 | 0.00000 |
|  | Recreational Space | 0.84 | 0.22 | 0.01100 |
|  | Found Neighborhood Space | -0.99 | 0.23 | 0.00100 |
|  | Park | 0.92 | 0.23 | 0.00300 |
|  | Memorial | 0.20 | 0.22 | 0.99900 |
|  | Playground | 1.20 | 0.22 | 0.00000 |
|  | Community Open Space | 1.48 | 0.23 | 0.00000 |
|  | Indoor Marketplace | 0.75 | 0.22 | 0.03100 |
|  | Waterfront | 0.81 | 0.22 | 0.01300 |
| Wildlife | Transport Facility | 0.93 | 0.22 | 0.00100 |
|  | Street | 0.21 | 0.21 | 0.99700 |
|  | Square | -0.55 | 0.21 | 0.28200 |
|  | Recreational Space | -0.72 | 0.21 | 0.03700 |
|  | Found Neighborhood Space | -0.28 | 0.22 | 0.98300 |
|  | Park | -1.22 | 0.22 | 0.00000 |
|  | Memorial | -1.24 | 0.21 | 0.00000 |
|  | Playground | -0.38 | 0.21 | 0.79300 |
|  | Community Open Space | 0.11 | 0.22 | 1.00000 |
|  | Indoor Marketplace | 0.90 | 0.21 | 0.00100 |
|  | Waterfront | 0.35 | 0.21 | 0.87100 |
| Lighting | Transport Facility | -1.08 | 0.21 | 0.00000 |
|  | Street | -0.79 | 0.20 | 0.00400 |
|  | Square | -1.38 | 0.20 | 0.00000 |
|  | Recreational Space | -1.23 | 0.20 | 0.00000 |
|  | Found Neighborhood Space | 0.71 | 0.21 | 0.03500 |
|  | Park | -1.00 | 0.21 | 0.00000 |
|  | Memorial | 0.32 | 0.20 | 0.91800 |
|  | Playground | -1.10 | 0.20 | 0.00000 |
|  | Community Open Space | -1.46 | 0.21 | 0.00000 |
|  | Indoor Marketplace | -1.51 | 0.20 | 0.00000 |
|  | Waterfront | -1.23 | 0.20 | 0.00000 |

**Playground (1 of 2)**

| Dependent Variable | Comparison Space Type | Difference | *SE* | *p* |
| --- | --- | --- | --- | --- |
| Comfort | Transport Facility | 1.25 | 0.20 | 0.00000 |
|  | Street | 0.88 | 0.19 | 0.00000 |
|  | Square | -0.17 | 0.20 | 1.00000 |
|  | Recreational Space | 0.00 | 0.20 | 1.00000 |
|  | Found Neighborhood Space | 1.84 | 0.20 | 0.00000 |
|  | Park | -0.53 | 0.20 | 0.25500 |
|  | Memorial | 0.93 | 0.20 | 0.00000 |
|  | Market | 0.80 | 0.20 | 0.00300 |
|  | Community Open Space | -0.44 | 0.20 | 0.55600 |
|  | Indoor Marketplace | 0.21 | 0.19 | 0.99500 |
|  | Waterfront | -0.20 | 0.19 | 0.99700 |
| Activity | Transport Facility | 0.92 | 0.20 | 0.00000 |
|  | Street | 0.21 | 0.19 | 0.99400 |
|  | Square | 0.32 | 0.20 | 0.90600 |
|  | Recreational Space | -0.09 | 0.20 | 1.00000 |
|  | Found Neighborhood Space | 2.81 | 0.20 | 0.00000 |
|  | Park | 0.64 | 0.20 | 0.06400 |
|  | Memorial | 2.08 | 0.20 | 0.00000 |
|  | Market | -0.37 | 0.20 | 0.76300 |
|  | Community Open Space | 1.17 | 0.20 | 0.00000 |
|  | Indoor Marketplace | 0.04 | 0.19 | 1.00000 |
|  | Waterfront | 0.19 | 0.19 | 0.99800 |
| Legibility | Transport Facility | 0.62 | 0.18 | 0.03100 |
|  | Street | 0.49 | 0.17 | 0.16300 |
|  | Square | -0.18 | 0.18 | 0.99700 |
|  | Recreational Space | -0.12 | 0.18 | 1.00000 |
|  | Found Neighborhood Space | 1.33 | 0.18 | 0.00000 |
|  | Park | 0.20 | 0.18 | 0.99600 |
|  | Memorial | 1.32 | 0.18 | 0.00000 |
|  | Market | 1.19 | 0.18 | 0.00000 |
|  | Community Open Space | -0.01 | 0.18 | 1.00000 |
|  | Indoor Marketplace | 0.37 | 0.17 | 0.59100 |
|  | Waterfront | -0.08 | 0.18 | 1.00000 |
| Enclosure | Transport Facility | -0.31 | 0.21 | 0.95400 |
|  | Street | -0.46 | 0.20 | 0.51700 |
|  | Square | 0.26 | 0.21 | 0.98300 |
|  | Recreational Space | 0.03 | 0.21 | 1.00000 |
|  | Found Neighborhood Space | -0.77 | 0.21 | 0.01600 |
|  | Park | -0.10 | 0.21 | 1.00000 |
|  | Memorial | -0.51 | 0.21 | 0.38400 |
|  | Market | -1.24 | 0.21 | 0.00000 |
|  | Community Open Space | 0.35 | 0.21 | 0.90300 |
|  | Indoor Marketplace | -0.14 | 0.20 | 1.00000 |
|  | Waterfront | 0.29 | 0.21 | 0.95800 |

**Playground (2 of 2)**

| Dependent Variable | Comparison Space Type | Difference | *SE* | *p* |
| --- | --- | --- | --- | --- |
| Complexity | Transport Facility | 0.04 | 0.19 | 1.00000 |
|  | Street | -0.64 | 0.19 | 0.03100 |
|  | Square | -0.01 | 0.19 | 1.00000 |
|  | Recreational Space | 0.56 | 0.19 | 0.14900 |
|  | Found Neighborhood Space | 1.62 | 0.20 | 0.00000 |
|  | Park | -0.06 | 0.20 | 1.00000 |
|  | Memorial | 0.34 | 0.19 | 0.82800 |
|  | Market | -0.92 | 0.19 | 0.00000 |
|  | Community Open Space | 0.30 | 0.20 | 0.93700 |
|  | Indoor Marketplace | -0.83 | 0.19 | 0.00100 |
|  | Waterfront | -0.44 | 0.19 | 0.46600 |
| Crime Potential | Transport Facility | -1.32 | 0.22 | 0.00000 |
|  | Street | -0.76 | 0.21 | 0.02000 |
|  | Square | -0.04 | 0.22 | 1.00000 |
|  | Recreational Space | -0.37 | 0.22 | 0.87600 |
|  | Found Neighborhood Space | -2.19 | 0.22 | 0.00000 |
|  | Park | -0.29 | 0.22 | 0.98000 |
|  | Memorial | -1.00 | 0.22 | 0.00000 |
|  | Market | -1.20 | 0.22 | 0.00000 |
|  | Community Open Space | 0.28 | 0.22 | 0.98400 |
|  | Indoor Marketplace | -0.46 | 0.21 | 0.59600 |
|  | Waterfront | -0.40 | 0.21 | 0.79500 |
| Wildlife | Transport Facility | 1.31 | 0.21 | 0.00000 |
|  | Street | 0.59 | 0.20 | 0.13100 |
|  | Square | -0.17 | 0.21 | 1.00000 |
|  | Recreational Space | -0.34 | 0.21 | 0.90000 |
|  | Found Neighborhood Space | 0.11 | 0.21 | 1.00000 |
|  | Park | -0.83 | 0.21 | 0.00500 |
|  | Memorial | -0.85 | 0.21 | 0.00200 |
|  | Market | 0.38 | 0.21 | 0.79300 |
|  | Community Open Space | 0.49 | 0.21 | 0.47800 |
|  | Indoor Marketplace | 1.28 | 0.20 | 0.00000 |
|  | Waterfront | 0.74 | 0.20 | 0.01800 |
| Lighting | Transport Facility | 0.03 | 0.20 | 1.00000 |
|  | Street | 0.31 | 0.19 | 0.90000 |
|  | Square | -0.27 | 0.20 | 0.97000 |
|  | Recreational Space | -0.13 | 0.20 | 1.00000 |
|  | Found Neighborhood Space | 1.81 | 0.20 | 0.00000 |
|  | Park | 0.11 | 0.20 | 1.00000 |
|  | Memorial | 1.42 | 0.20 | 0.00000 |
|  | Market | 1.10 | 0.20 | 0.00000 |
|  | Community Open Space | -0.36 | 0.20 | 0.84900 |
|  | Indoor Marketplace | -0.41 | 0.19 | 0.62400 |
|  | Waterfront | -0.12 | 0.20 | 1.00000 |

**Community Open Space (1 of 2)**

| Dependent Variable | Comparison Space Type | Difference | *SE* | *p* |
| --- | --- | --- | --- | --- |
| Comfort | Transport Facility | 1.69 | 0.21 | 0.00000 |
|  | Street | 1.33 | 0.20 | 0.00000 |
|  | Square | 0.28 | 0.21 | 0.97300 |
|  | Recreational Space | 0.45 | 0.21 | 0.59900 |
|  | Found Neighborhood Space | 2.28 | 0.21 | 0.00000 |
|  | Park | -0.09 | 0.21 | 1.00000 |
|  | Memorial | 1.37 | 0.21 | 0.00000 |
|  | Market | 1.24 | 0.21 | 0.00000 |
|  | Playground | 0.44 | 0.20 | 0.55600 |
|  | Indoor Marketplace | 0.66 | 0.20 | 0.06100 |
|  | Waterfront | 0.24 | 0.21 | 0.99100 |
| Activity | Transport Facility | -0.25 | 0.21 | 0.98900 |
|  | Street | -0.96 | 0.20 | 0.00000 |
|  | Square | -0.85 | 0.21 | 0.00300 |
|  | Recreational Space | -1.26 | 0.21 | 0.00000 |
|  | Found Neighborhood Space | 1.65 | 0.21 | 0.00000 |
|  | Park | -0.53 | 0.21 | 0.33700 |
|  | Memorial | 0.91 | 0.21 | 0.00100 |
|  | Market | -1.54 | 0.21 | 0.00000 |
|  | Playground | -1.17 | 0.20 | 0.00000 |
|  | Indoor Marketplace | -1.12 | 0.20 | 0.00000 |
|  | Waterfront | -0.98 | 0.20 | 0.00000 |
| Legibility | Transport Facility | 0.63 | 0.19 | 0.04500 |
|  | Street | 0.51 | 0.18 | 0.20600 |
|  | Square | -0.17 | 0.19 | 0.99900 |
|  | Recreational Space | -0.11 | 0.19 | 1.00000 |
|  | Found Neighborhood Space | 1.34 | 0.19 | 0.00000 |
|  | Park | 0.21 | 0.19 | 0.99500 |
|  | Memorial | 1.33 | 0.19 | 0.00000 |
|  | Market | 1.20 | 0.19 | 0.00000 |
|  | Playground | 0.01 | 0.18 | 1.00000 |
|  | Indoor Marketplace | 0.39 | 0.18 | 0.63300 |
|  | Waterfront | -0.07 | 0.19 | 1.00000 |
| Enclosure | Transport Facility | -0.65 | 0.22 | 0.13500 |
|  | Street | -0.80 | 0.22 | 0.01100 |
|  | Square | -0.08 | 0.22 | 1.00000 |
|  | Recreational Space | -0.31 | 0.22 | 0.95900 |
|  | Found Neighborhood Space | -1.12 | 0.23 | 0.00000 |
|  | Park | -0.45 | 0.22 | 0.69000 |
|  | Memorial | -0.85 | 0.22 | 0.00600 |
|  | Market | -1.58 | 0.22 | 0.00000 |
|  | Playground | -0.35 | 0.21 | 0.90300 |
|  | Indoor Marketplace | -0.48 | 0.22 | 0.52100 |
|  | Waterfront | -0.05 | 0.22 | 1.00000 |

**Community Open Space (2 of 2)**

| Dependent Variable | Comparison Space Type | Difference | *SE* | *p* |
| --- | --- | --- | --- | --- |
| Complexity | Transport Facility | -0.26 | 0.21 | 0.98100 |
|  | Street | -0.94 | 0.20 | 0.00000 |
|  | Square | -0.31 | 0.20 | 0.93800 |
|  | Recreational Space | 0.26 | 0.20 | 0.98300 |
|  | Found Neighborhood Space | 1.32 | 0.21 | 0.00000 |
|  | Park | -0.36 | 0.21 | 0.84100 |
|  | Memorial | 0.04 | 0.20 | 1.00000 |
|  | Market | -1.22 | 0.20 | 0.00000 |
|  | Playground | -0.30 | 0.20 | 0.93700 |
|  | Indoor Marketplace | -1.13 | 0.20 | 0.00000 |
|  | Waterfront | -0.74 | 0.20 | 0.01300 |
| Crime Potential | Transport Facility | -1.60 | 0.23 | 0.00000 |
|  | Street | -1.04 | 0.22 | 0.00000 |
|  | Square | -0.32 | 0.23 | 0.96700 |
|  | Recreational Space | -0.65 | 0.23 | 0.17900 |
|  | Found Neighborhood Space | -2.48 | 0.24 | 0.00000 |
|  | Park | -0.57 | 0.23 | 0.39100 |
|  | Memorial | -1.28 | 0.23 | 0.00000 |
|  | Market | -1.48 | 0.23 | 0.00000 |
|  | Playground | -0.28 | 0.22 | 0.98400 |
|  | Indoor Marketplace | -0.74 | 0.23 | 0.05300 |
|  | Waterfront | -0.68 | 0.23 | 0.11900 |
| Wildlife | Transport Facility | 0.82 | 0.22 | 0.01300 |
|  | Street | 0.10 | 0.21 | 1.00000 |
|  | Square | -0.66 | 0.22 | 0.10500 |
|  | Recreational Space | -0.83 | 0.22 | 0.01000 |
|  | Found Neighborhood Space | -0.39 | 0.22 | 0.85700 |
|  | Park | -1.33 | 0.22 | 0.00000 |
|  | Memorial | -1.35 | 0.22 | 0.00000 |
|  | Market | -0.11 | 0.22 | 1.00000 |
|  | Playground | -0.49 | 0.21 | 0.47800 |
|  | Indoor Marketplace | 0.79 | 0.22 | 0.01400 |
|  | Waterfront | 0.24 | 0.22 | 0.99300 |
| Lighting | Transport Facility | 0.38 | 0.21 | 0.82200 |
|  | Street | 0.67 | 0.21 | 0.05200 |
|  | Square | 0.09 | 0.21 | 1.00000 |
|  | Recreational Space | 0.23 | 0.21 | 0.99500 |
|  | Found Neighborhood Space | 2.17 | 0.21 | 0.00000 |
|  | Park | 0.46 | 0.21 | 0.56900 |
|  | Memorial | 1.78 | 0.21 | 0.00000 |
|  | Market | 1.46 | 0.21 | 0.00000 |
|  | Playground | 0.36 | 0.20 | 0.84900 |
|  | Indoor Marketplace | -0.05 | 0.21 | 1.00000 |
|  | Waterfront | 0.23 | 0.21 | 0.99300 |

**Indoor Marketplace (1 of 2)**

| Dependent Variable | Comparison Space Type | Difference | *SE* | *p* |
| --- | --- | --- | --- | --- |
| Comfort | Transport Facility | 1.04 | 0.20 | 0.00000 |
|  | Street | 0.67 | 0.19 | 0.02800 |
|  | Square | -0.38 | 0.20 | 0.75800 |
|  | Recreational Space | -0.21 | 0.20 | 0.99600 |
|  | Found Neighborhood Space | 1.63 | 0.20 | 0.00000 |
|  | Park | -0.74 | 0.20 | 0.01300 |
|  | Memorial | 0.71 | 0.20 | 0.01800 |
|  | Market | 0.58 | 0.20 | 0.12200 |
|  | Playground | -0.21 | 0.19 | 0.99500 |
|  | Community Open Space | -0.66 | 0.20 | 0.06100 |
|  | Waterfront | -0.41 | 0.20 | 0.61200 |
| Activity | Transport Facility | 0.87 | 0.20 | 0.00100 |
|  | Street | 0.17 | 0.19 | 0.99900 |
|  | Square | 0.27 | 0.20 | 0.96700 |
|  | Recreational Space | -0.14 | 0.20 | 1.00000 |
|  | Found Neighborhood Space | 2.77 | 0.20 | 0.00000 |
|  | Park | 0.60 | 0.20 | 0.12300 |
|  | Memorial | 2.04 | 0.20 | 0.00000 |
|  | Market | -0.41 | 0.20 | 0.62400 |
|  | Playground | -0.04 | 0.19 | 1.00000 |
|  | Community Open Space | 1.12 | 0.20 | 0.00000 |
|  | Waterfront | 0.14 | 0.20 | 1.00000 |
| Legibility | Transport Facility | 0.25 | 0.18 | 0.97100 |
|  | Street | 0.12 | 0.18 | 1.00000 |
|  | Square | -0.56 | 0.18 | 0.08400 |
|  | Recreational Space | -0.49 | 0.18 | 0.21700 |
|  | Found Neighborhood Space | 0.96 | 0.18 | 0.00000 |
|  | Park | -0.18 | 0.18 | 0.99800 |
|  | Memorial | 0.94 | 0.18 | 0.00000 |
|  | Market | 0.82 | 0.18 | 0.00000 |
|  | Playground | -0.37 | 0.17 | 0.59100 |
|  | Community Open Space | -0.39 | 0.18 | 0.63300 |
|  | Waterfront | -0.46 | 0.18 | 0.29300 |
| Enclosure | Transport Facility | -0.17 | 0.21 | 1.00000 |
|  | Street | -0.32 | 0.20 | 0.92400 |
|  | Square | 0.40 | 0.21 | 0.75300 |
|  | Recreational Space | 0.17 | 0.21 | 1.00000 |
|  | Found Neighborhood Space | -0.64 | 0.22 | 0.12400 |
|  | Park | 0.04 | 0.21 | 1.00000 |
|  | Memorial | -0.37 | 0.21 | 0.83800 |
|  | Market | -1.10 | 0.21 | 0.00000 |
|  | Playground | 0.14 | 0.20 | 1.00000 |
|  | Community Open Space | 0.48 | 0.22 | 0.52100 |
|  | Waterfront | 0.43 | 0.21 | 0.63700 |

**Indoor Marketplace (2 of 2)**

| Dependent Variable | Comparison Space Type | Difference | *SE* | *p* |
| --- | --- | --- | --- | --- |
| Complexity | Transport Facility | 0.87 | 0.20 | 0.00100 |
|  | Street | 0.19 | 0.19 | 0.99700 |
|  | Square | 0.83 | 0.19 | 0.00100 |
|  | Recreational Space | 1.39 | 0.19 | 0.00000 |
|  | Found Neighborhood Space | 2.45 | 0.20 | 0.00000 |
|  | Park | 0.77 | 0.20 | 0.00600 |
|  | Memorial | 1.18 | 0.19 | 0.00000 |
|  | Market | -0.09 | 0.19 | 1.00000 |
|  | Playground | 0.83 | 0.19 | 0.00100 |
|  | Community Open Space | 1.13 | 0.20 | 0.00000 |
|  | Waterfront | 0.39 | 0.19 | 0.64700 |
| Crime Potential | Transport Facility | -0.86 | 0.22 | 0.00700 |
|  | Street | -0.30 | 0.21 | 0.96300 |
|  | Square | 0.42 | 0.22 | 0.75300 |
|  | Recreational Space | 0.09 | 0.22 | 1.00000 |
|  | Found Neighborhood Space | -1.74 | 0.22 | 0.00000 |
|  | Park | 0.17 | 0.22 | 1.00000 |
|  | Memorial | -0.55 | 0.22 | 0.34500 |
|  | Market | -0.75 | 0.22 | 0.03100 |
|  | Playground | 0.46 | 0.21 | 0.59600 |
|  | Community Open Space | 0.74 | 0.23 | 0.05300 |
|  | Waterfront | 0.06 | 0.22 | 1.00000 |
| Wildlife | Transport Facility | 0.03 | 0.21 | 1.00000 |
|  | Street | -0.69 | 0.20 | 0.03800 |
|  | Square | -1.45 | 0.21 | 0.00000 |
|  | Recreational Space | -1.62 | 0.21 | 0.00000 |
|  | Found Neighborhood Space | -1.17 | 0.21 | 0.00000 |
|  | Park | -2.11 | 0.21 | 0.00000 |
|  | Memorial | -2.13 | 0.21 | 0.00000 |
|  | Market | -0.90 | 0.21 | 0.00100 |
|  | Playground | -1.28 | 0.20 | 0.00000 |
|  | Community Open Space | -0.79 | 0.22 | 0.01400 |
|  | Waterfront | -0.54 | 0.21 | 0.26000 |
| Lighting | Transport Facility | 0.43 | 0.20 | 0.59800 |
|  | Street | 0.72 | 0.20 | 0.01200 |
|  | Square | 0.14 | 0.20 | 1.00000 |
|  | Recreational Space | 0.28 | 0.20 | 0.96400 |
|  | Found Neighborhood Space | 2.22 | 0.21 | 0.00000 |
|  | Park | 0.52 | 0.20 | 0.32200 |
|  | Memorial | 1.83 | 0.20 | 0.00000 |
|  | Market | 1.51 | 0.20 | 0.00000 |
|  | Playground | 0.41 | 0.19 | 0.62400 |
|  | Community Open Space | 0.05 | 0.21 | 1.00000 |
|  | Waterfront | 0.29 | 0.20 | 0.95300 |

**Waterfront (1 of 2)**

| Dependent Variable | Comparison Space Type | Difference | *SE* | *p* |
| --- | --- | --- | --- | --- |
| Comfort | Transport Facility | 1.45 | 0.20 | 0.00000 |
|  | Street | 1.08 | 0.19 | 0.00000 |
|  | Square | 0.04 | 0.20 | 1.00000 |
|  | Recreational Space | 0.20 | 0.20 | 0.99700 |
|  | Found Neighborhood Space | 2.04 | 0.20 | 0.00000 |
|  | Park | -0.33 | 0.20 | 0.90300 |
|  | Memorial | 1.13 | 0.20 | 0.00000 |
|  | Market | 1.00 | 0.20 | 0.00000 |
|  | Playground | 0.20 | 0.19 | 0.99700 |
|  | Community Open Space | -0.24 | 0.21 | 0.99100 |
|  | Indoor Marketplace | 0.41 | 0.20 | 0.61200 |
| Activity | Transport Facility | 0.73 | 0.20 | 0.01700 |
|  | Street | 0.02 | 0.19 | 1.00000 |
|  | Square | 0.13 | 0.20 | 1.00000 |
|  | Recreational Space | -0.28 | 0.20 | 0.96200 |
|  | Found Neighborhood Space | 2.63 | 0.20 | 0.00000 |
|  | Park | 0.45 | 0.20 | 0.53200 |
|  | Memorial | 1.89 | 0.20 | 0.00000 |
|  | Market | -0.56 | 0.20 | 0.17700 |
|  | Playground | -0.19 | 0.19 | 0.99800 |
|  | Community Open Space | 0.98 | 0.20 | 0.00000 |
|  | Indoor Marketplace | -0.14 | 0.20 | 1.00000 |
| Legibility | Transport Facility | 0.70 | 0.18 | 0.00700 |
|  | Street | 0.58 | 0.18 | 0.05100 |
|  | Square | -0.10 | 0.18 | 1.00000 |
|  | Recreational Space | -0.03 | 0.18 | 1.00000 |
|  | Found Neighborhood Space | 1.41 | 0.19 | 0.00000 |
|  | Park | 0.28 | 0.18 | 0.93700 |
|  | Memorial | 1.40 | 0.18 | 0.00000 |
|  | Market | 1.27 | 0.18 | 0.00000 |
|  | Playground | 0.08 | 0.18 | 1.00000 |
|  | Community Open Space | 0.07 | 0.19 | 1.00000 |
|  | Indoor Marketplace | 0.46 | 0.18 | 0.29300 |
| Enclosure | Transport Facility | -0.60 | 0.21 | 0.18500 |
|  | Street | -0.75 | 0.21 | 0.01600 |
|  | Square | -0.03 | 0.21 | 1.00000 |
|  | Recreational Space | -0.26 | 0.21 | 0.98600 |
|  | Found Neighborhood Space | -1.07 | 0.22 | 0.00000 |
|  | Park | -0.40 | 0.22 | 0.79700 |
|  | Memorial | -0.80 | 0.21 | 0.00900 |
|  | Market | -1.53 | 0.21 | 0.00000 |
|  | Playground | -0.29 | 0.21 | 0.95800 |
|  | Community Open Space | 0.05 | 0.22 | 1.00000 |
|  | Indoor Marketplace | -0.43 | 0.21 | 0.63700 |

**Waterfront (2 of 2)**

| Dependent Variable | Comparison Space Type | Difference | *SE* | *p* |
| --- | --- | --- | --- | --- |
| Complexity | Transport Facility | 0.47 | 0.20 | 0.40800 |
|  | Street | -0.20 | 0.19 | 0.99600 |
|  | Square | 0.43 | 0.20 | 0.54000 |
|  | Recreational Space | 1.00 | 0.20 | 0.00000 |
|  | Found Neighborhood Space | 2.06 | 0.20 | 0.00000 |
|  | Park | 0.38 | 0.20 | 0.76600 |
|  | Memorial | 0.78 | 0.20 | 0.00400 |
|  | Market | -0.48 | 0.19 | 0.35500 |
|  | Playground | 0.44 | 0.19 | 0.46600 |
|  | Community Open Space | 0.74 | 0.20 | 0.01300 |
|  | Indoor Marketplace | -0.39 | 0.19 | 0.64700 |
| Crime Potential | Transport Facility | -0.92 | 0.22 | 0.00300 |
|  | Street | -0.36 | 0.22 | 0.88100 |
|  | Square | 0.36 | 0.22 | 0.90000 |
|  | Recreational Space | 0.03 | 0.22 | 1.00000 |
|  | Found Neighborhood Space | -1.80 | 0.23 | 0.00000 |
|  | Park | 0.11 | 0.23 | 1.00000 |
|  | Memorial | -0.61 | 0.22 | 0.20600 |
|  | Market | -0.81 | 0.22 | 0.01300 |
|  | Playground | 0.40 | 0.21 | 0.79500 |
|  | Community Open Space | 0.68 | 0.23 | 0.11900 |
|  | Indoor Marketplace | -0.06 | 0.22 | 1.00000 |
| Wildlife | Transport Facility | 0.57 | 0.21 | 0.23500 |
|  | Street | -0.14 | 0.21 | 1.00000 |
|  | Square | -0.91 | 0.21 | 0.00100 |
|  | Recreational Space | -1.07 | 0.21 | 0.00000 |
|  | Found Neighborhood Space | -0.63 | 0.22 | 0.13400 |
|  | Park | -1.57 | 0.21 | 0.00000 |
|  | Memorial | -1.59 | 0.21 | 0.00000 |
|  | Market | -0.35 | 0.21 | 0.87100 |
|  | Playground | -0.74 | 0.20 | 0.01800 |
|  | Community Open Space | -0.24 | 0.22 | 0.99300 |
|  | Indoor Marketplace | 0.54 | 0.21 | 0.26000 |
| Lighting | Transport Facility | 0.15 | 0.20 | 1.00000 |
|  | Street | 0.44 | 0.20 | 0.53800 |
|  | Square | -0.15 | 0.20 | 1.00000 |
|  | Recreational Space | 0.00 | 0.20 | 1.00000 |
|  | Found Neighborhood Space | 1.93 | 0.21 | 0.00000 |
|  | Park | 0.23 | 0.21 | 0.99400 |
|  | Memorial | 1.55 | 0.20 | 0.00000 |
|  | Market | 1.23 | 0.20 | 0.00000 |
|  | Playground | 0.12 | 0.20 | 1.00000 |
|  | Community Open Space | -0.23 | 0.21 | 0.99300 |
|  | Indoor Marketplace | -0.29 | 0.20 | 0.95300 |
